# Positive impact of hydroponics and artificial light on yield and quality of wheat

**DOI:** 10.1038/s41598-025-16204-0

**Published:** 2025-08-21

**Authors:** Simona Bassu, Sebastian Eichelsbacher, Francesco Giunta, Rosella Motzo, Corinna Dawid, Martina Gastl, Michael Schloter, Katharina A. Scherf, Stefan Hör, Yuri Pinheiro Alves De Souza, Stefanie Schulz, Timo D. Stark, Volker Mohler, Senthold Asseng

**Affiliations:** 1 Chair of Digital Agriculture, Department of Life Science Engineering, HEF World Agricultural Systems Center, School of Life Sciences, Technical University of Munich, Freising, Germany; 2 Department of Agricultural Sciences, University of Sassari, Sassari, Italy; 3 TUM School of Life Sciences, Chemosensory Food Systems, Technical University of Munich, Freising, Germany; 4 Research Center Weihenstephan for Brewing and Food Quality, Technical University of Munich, Freising, Germany; 5 Present Address: Research Unit Comparative Microbiome Analysis, Helmholtz Zentrum München, Neuherberg, Germany; 6 Leibniz Institute for Food Systems Biology at the Technical University of Munich, Freising, Germany; 7 TUM School of Life Sciences, Professorship of Food Biopolymer Systems, Technical University of Munich, Freising, Germany; 8 TUM School of Life Sciences, Food Chemistry and Molecular Sensory Science, Technical University of Munich, Freising, Germany; 9 Bavarian State Research Center for Agriculture, Freising, Germany; 10 TUM School of Life Sciences, Professorship of Environmental Microbiology, HEF World Agricultural Systems Center, Technical University of Munich, Freising, Germany

**Keywords:** Wheat, Yield, Nutritional and baking quality, Microbiome, Metabolites, Gliadins, Physiology, Plant sciences

## Abstract

Growing crops in controlled-environment indoor farming systems offers new ways of producing high-yield, pesticide-free, environmental-friendly food. However, it replaces soil with hydroponics and the sun with LED lights. Compared with the field, wheat grown indoors showed a much higher yield potential and bread-making quality parameters. Many mineral concentrations were higher due to the unrestricted water supply and nutrients in hydroponics. However, concentrations declined with increasing yields. The microbiome richness inside the grains of wheat grown without soil indoors was still within the range of wheat grown in the field. However, taxa were different among cultivars and treatments. There were differences in the presence of undefined secondary metabolites between indoor and outdoor wheat and across the indoor experiments. Regardless of the growing environment, immunoreactive proteins were present. Indoor-grown wheat had a higher share of ω5-gliadins but lower shares of γ-gliadins and low‐molecular‐weight glutenin subunits, which may affect the gluten protein immunoreactive potential for individuals with wheat-related disorders (allergy and celiac disease). Growing wheat without soil and sunlight indoors can produce high-yielding, high-quality grains. However, the food quality and health aspects associated with gluten proteins might deteriorate with a further, theoretically possible, yield increase in a controlled growing environment.

## Introduction

Wheat (*Triticum aestivum* L.) is the most widely cultivated, most traded crop and is, therefore, critical in ensuring global food security. While the demand for wheat supply is expected to increase with the continuous growth of the world population to more than 9 billion by 2050, traditional agriculture, strained by climate variability, increasing temperatures, and droughts, will be challenged to meet this demand^1–3^. Vertical farming, where crops are grown without soil using artificial light in stacked layers, is a promising and innovative technology of advanced and sustainable agriculture, which, despite the current high energy demand and costs, offers new ways of highly productive, pesticide-free, environmental-friendly food production. In a recent study, Asseng et al.^4^ simulated that controlled and optimized growth conditions in vertically stacked layers enable very high yields per harvest and multiple wheat harvests per year. This results in a 6000 times higher average yield than the field, per hectare, and year in a vertical farm with 100 stacked layers and a crop life cycle of 70 days from seeding to harvest.

While field experiments with wheat have shown that increasing yields tend to reduce grain protein concentration^5^ and some micronutrients^6^, the impact of very high yields grown under controlled environment conditions on grain quality is still unknown. Several studies have indicated a decline in wheat grain quality with increasing grain yields under enriched atmospheric CO_2_ concentration^6–11^. In particular, Hogy et al.^12^ reported that grain size distribution was shifted to smaller grains, lower protein concentration, including gluten and gliadins, and minerals like manganese and iron decreased. However, the same authors found that other grain quality traits important for bread-making quality, such as starch, were unaffected by elevated atmospheric CO_2_ concentration. In contrast to elevated CO_2_ concentrations, specific light spectra might improve crop growth, yield^13^, and grain quality of wheat^14,15^.

While several studies reported the combined impacts of individual light characteristics and elevated atmospheric CO_2_ concentration on wheat yield and grain quality, the combined impact of growing wheat under artificial light without soil compared to field-grown wheat has never been addressed. This also includes questions about the possible impact of secondary metabolite patterns and concentrations of immunoreactive proteins associated with wheat-related disorders^16^. Effects on grain quality are likely to be expected. A sterile growth environment with no soils may induce shifts in root-environment interactions with pronounced effects on the plant`s phenotype. Moreover, the role of microbiota from soil, which acts as a reservoir for the plant-associated microbiome and drives plant phenotypes to a large extent^17^, is missing.

Here, we explore the impact of a soilless indoor cultivation system, artificial light, and varying indoor-yield environments on yield, grain quality for bread-making, the microbiome inside grains of wheat, metabolites, and immunoreactive grain protein components and compare the results with those of modern bread-wheat cultivars grown in the field. We postulate differences in major wheat properties between plants grown under field and indoor conditions as a matter of the different abiotic conditions, although comparable wheat cultivars based on their genetic background are included in the study.

## Materials and methods

### Field experiments

The wheat (*Triticum aestivum* L.) cultivar Apogee was sown at a rate of 350 seeds m^−2^ on 21 December 2021 together with seven modern spring wheat cultivars representative of spring wheat cultivars currently grown for the bread industry at the experimental station of Sassari (41 °N; 8 °E; 80 m elevation; Sardinia, Italy) under rainfed conditions, in the absence of nutrient limitations and with pests and diseases chemically controlled. The climate of the location is typically Mediterranean, with a long-term annual average rainfall of 557 ± 131 mm, mainly concentrated between October and April. Thermal conditions showed the typical pattern of a Mediterranean climate. Winters are mild, with minimum temperatures rarely falling below 0 °C. The average monthly temperatures are not lower than 9.9 °C while increasing above 17.0 °C from May onwards.

The soil was a sandy clay loam of a maximum depth of about 0.6–0.7 m overlying a limestone bedrock (Xerochrepts). Plots consisted of 8 rows 8.4 m long and a between-row distance of 0.15 m, totaling 10 m^2^, and were arranged randomly with three replications for cultivar Apogee. The other seven cultivars grown in the field were used to represent some of the genotypic variability within modern bread wheat cultivars. As the genotypic variability is low and no significant differences in the phenotypic variability were observed, the seven cultivars were considered as replicates for further analysis and compared with the cultivar Apogee grown in the field and the three indoor treatments. Means were compared using the Welch’s *t*-test^18^, to take into account any difference in variance between treatments. *P*-values of key parameters obtained from Welch’s *t*-test comparing pairs of treatments are shown in the Supplementary Table S1.

Nitrogen and phosphorus fertilizer were applied at sowing at a rate of 100 kg ha^−1^ of nitrogen and 42 kg ha^−1^ of phosphorus. In both years, anthesis and physiological maturity (yellow peduncle) were recorded when observed in more than 50% of each plot. Grain yield was obtained per plot using mechanical harvesting. Grain weight and grain yield are expressed at 0% moisture. The number of grains m^−2^ was calculated as the ratio between grain yield and average grain weight. Meteorological records (daily values of incoming solar radiation, maximum and minimum temperatures, and total rainfall) were recorded at a meteorological station located in the field.

### Indoor experiments

The wheat (*Triticum aestivum* L.) cultivar Apogee was grown in three indoor experiments with fully controlled environmental conditions at the TUM Plant Technology Center at Technical University of Munich in Freising, Germany. Cultivar Apogee is a high-yielding, double dwarf, early cultivar that has been developed by the National Aeronautics and Space Administration (NASA) for controlled environments growing conditions^19^. This cultivar was then compared with other modern wheat cultivars in the field under the same growing conditions to evaluate possible differences of cultivar Apogee and current modern cultivars. The cultivar Apogee was obtained from the Utah Agricultural Experiment Station at Utah State University (USU), where it was released in 1996 in cooperation with NASA. The three indoor experiments mainly differed in the light intensity and CO_2_ concentration (Table 1). Experiment 1, called hereafter ‘low’ (L) -yielding experiment, was performed using atmospheric CO_2_ concentrations (about 419 ppm) and, on average, 500 μmol m^−2^ s^−1^ of Photosynthetic Photon Flux Density (PPFD). Experiment 2, called hereafter ‘medium’ (M) -yielding experiment, was characterized by atmospheric CO_2_ concentrations and 700 μmol m^−2^ s^−1^ of PPFD. Experiment 3, called the ‘high’ (H) -yielding experiment, was performed with an elevated CO_2_ concentration of 1000 ppm and 1000 μmol m^−2^ s^−1^ of PPFD. Table 1 shows the environmental parameters of the three indoor experiments.

**Table 1:** Summary of the environmental conditions and the cultivars grown for the field experiment (F) and the three indoor experiments, including low (L), medium (M), and high (H) -yielding wheat growth conditions.

Parameters	F	L	M	H
Average daily temperature above 0 °C (°C)^a^	16	24	21	22
CO_2_ concentration (ppm)	417^b^	419^c^	419^c^	1000
Average PAR light intensity^a^ (mol m^−2^ d^−1^)	34^d^	34	39	54
Cumulative PAR light^a^ (mol m^−2^)	5714^e^	2524	2996	4086
Days from sowing to maturity	174	74	76	76
Thermal time from sowing to maturity (degree days)	2716	1752	1605	1696
Photoperiod during the growth phase (h d^−1^)	12	24	22	22
Harvest date	Jun-22	Apr-23	Apr-24	Feb-24
Cultivar	Albatros, Altamira	Apogee	Apogee	Apogee
	Apogee, Ascott			
	Bologna, Cougar			
	Torril, Zitnica			

^a^During the growing season, i.e. from sowing date to maturity.

^b^Based on global atmospheric CO_2_ concentrations in 2022 by NOAA^20^. Air was constantly replaced in the chambers with outdoors air and there could be some fluctuation around the average value.

^c^Based on global atmospheric CO_2_ concentrations in 2023 by NOAA^21^. Air was constantly replaced in the chambers with outdoors air and there could be some fluctuation around the average value.

^d^7.4 MJ m^−2^ d^−1^ (2.5 KWh m^−2^ d^−1^).

^e^1256 MJ m^−2^ (348.8 KWh m^−2^ d^−1^).

While the L and M experiments were characterized by the same temperature during the day and night (24 and 21 °C, respectively), in the H experiment, the temperature was 23 °C during the day and 20 °C at night. All crops in these experiments were grown with a photoperiod that ranged from 22 to 24 h d^−1^ of light, with a full spectrum from LED lights. The LED © Polyklima True Daylight PLUS was used as a full spectrum for the L and M experiments. In M and H experiments, further LEDs provided specific color spectra. In M, a red peak around 660 nm and a blue peak around 450 nm added color spectra. The spectrum for H is described in Jákli et al.^22^. Relative humidity levels were kept near 60–70% for all experiments. In all experiments, rockwool plugs were used to germinate the plants in moist conditions and provide holding structures for the roots. The roots grow through the plug to reach the nutrient solution.

The surface area of the experiments was 2.12 × 1.3 m (2.8 m^2^) for the L experiment on two layers, 2.12 × 1.3 m (2.8 m^2^) for the M experiment, and 2.25 × 1.3 m (2.9 m^2^) for the H experiment. The canopy was assembled with individual trays of 0.18 m^2^ containing single 150 rockwool plugs (2.5 × 2.5 cm). The seeding density was 870 seeds per m^2^. Yield parameters were determined for individual trays (0.18 m^2^) to understand variations and border effects across the canopy. 32, 4 and 8 technical trays were considered as replicates for the L, M, H indoor experiments, respectively.

Trays were placed on grids with a height of 3 cm in a deep-water system. In the L experiment, an ebb-flood hydroponic system watered the plants regularly with a nutrient solution. A deep water hydroponic system was used with roots permanently immersed in a nutrient solution for the M and H experiments. An air compressor pushed air into the deep water system through an air stone. The nutrient solutions were based on the Hoagland protocol and prepared with highly concentrated stock solutions before being diluted with deionized water and added into the root zones. A target pH of 5.9 and an electric conductivity of EC 2 were kept for the nutrient solution preparation, while values changed later in the root zone. The grain is sourced from a selection of trays spread across the canopy. Samples of the L experiment also included border trays. The other two experiments excluded border trays from the analysis.

At physiological maturity, plants were separated into ears and remaining aboveground biomass. Aboveground biomass was oven-dried at 60 °C for 72 h. Ears were threshed with a laboratory thresher to obtain the grain weight and to calculate the harvest index (HI), which is the ratio of grain weight to total aboveground biomass per plant. Grain moisture was determined with a grain moisture analyzer. The grain number per m^2^ was calculated as the grain yield and weight ratio. After threshing and before grain analyses, the grains were stored in paper bags at room temperature and protected from light for later analysis. Each laboratory analysis was carried out twice, with a mean representing two technical replications of a sample.

### Grain quality

Standard grain quality analyses were performed to assess the grain quality (Table 2).

**Table 2:** Methods used for standard grain quality analysis.

Grain quality analysis	Method
Ash	VO (EG) 152/2009, Anhang III, M (1)
Falling number	ICC-Standard Nr. 107
Fat	VO (EG) 152/2009, Anhang III, H (1)
Fibre	VO (EG) 152/2009, Anhang III, I (1)
Gluten index	ICC Standard 158
Grain sizes	MEBAK R-110.22.011 [2016–03] (1)
Minerals (Ca, P, Na, Mg, K, Fe, Cu, Mn, Zn)	DIN EN 15510:2007 (1)
Mycotoxins (DON, OTA, ZEA, AflaB1, B2)	DIN EN 17194 2017-12 (1)
Protein	VO (EG) 152/2009, Anhang III, C (1)
Sedimentation	Zeleny ICC-Standard Nr. 116/1
Starch	VO (EG) 152/2009, Anhang III, L (1)
Water	VO (EG) 152/2009, Anhang III, A (1)
Wet gluten	ICC Standard 155

The results are given as a percentage of dry weight, as well as weight per single grain.

### Grain microbiome

#### DNA extraction

Surface sterilized seeds were ground using sterilized mortars and stored at − 20° until DNA extraction. Seed DNA was extracted following a Phenol/Chloroform/Isoamyl alcohol-based method^23^. The analysis was done using the Lysing Matrix E tubes (MP Biomedicals™, Germany). The bead beating was done using a TissueLyser II bead beater (QIAGEN®, Germany) at a frequency of 15 Hz for 2 min. Using the broad-range assay kit, a Qubit fluorometric system (Thermo Fisher Scientific, Germany) quantified the resulting DNA. The quality of the DNA was checked using the Nanodrop photometric system (Thermo Fisher Scientific, Germany) and by agarose gel electrophoresis. A blank control without seed material was processed parallel to exclude contaminations during DNA extraction.

#### Amplicon library preparation and sequencing

A metabarcoding targeting of the V3 and V4 region of the 16S rRNA gene was performed using chloroplast exclusion primers S-D-Bact-0335-a-S-17 (338f. -TCGTCGGCAGCGTCAGATGTGTATAAGAGACAGCADACTCCTACGGGAGGC) and S-D-Bact-0769-a-A-19 (789r-GTCTCGTGGGCTCGGAGATGTGTATAAGAGACAGATCCTGTTTGMTMCCCVCRC)^24^ with an overhang sequence at the 5’ end compatible with the Nextera® XT Index Kit. The used primers reduced any overamplification of chloroplast sequences. PCR amplification was done using 20 ng of template DNA, and negative controls without DNA template were processed alongside. Each PCR reaction consisted of 25 µL containing 12.5 µL NEB Next High-Fidelity Master Mix (Thermo Fisher Scientific, Germany), 0.5 µL of each primer at 10 pmol µl^−1^, 2.5 µL of 3% BSA, 1µl of 5 ng µL^−1^ diluted DNA, and 8 µL of DEPC treated water. The thermal profile was 98 °C for 1 min, followed by 30 cycles of 98 °C for 10 s, 60 °C for 30 s, and 72 °C for 30 s, ended by a final extension of 72 °C for 5 min. Samples were indexed using the Nextera® XT Index Kit v2 (Illumina, USA) and purified with MagSi-NGSprep Plus Beads (ratio 0, 8 beads: 1 sample) according to the manufacturer’s protocol. Quality assessment was done via Fragment Analyser (Agilent, Germany). High-quality DNA was diluted to 4 nM and sequenced on Illumina MiSeq using a MiSeq Reagent v3 (600 Cycle) kit. Five pM 20% PhiX had been loaded alongside the samples.

#### Sequence processing

After sequencing, samples were uploaded to the European Galaxy server (https://usegalaxy.eu). A Cutadpat^25^ tool was used to remove adapters, and the quality of the reads was assessed using FastQC^26^. Forward readings with a quality score below 30 and reverse readings with a quality score below 20 were removed. For further analysis, dada2 version 1.16^27^ was used. The plotQualityProfile option was used to determine the trimming parameters, which were set to 280 bp for the forward and 220 bp for the reverse reads. The following steps included calculating error rates and sample inference, merging reads, and removing chimeric sequences. Taxonomy was assigned using assignTaxonomy and addSpecies functions, aligning the ASVs against the Silva database^28^ version 138.

Plots and statistical analysis were conducted in R version 4.2.2^29^ using the packages phyloseq version 1.42.0 [11] and vegan^30^ version 4.0.5. Before analysis, all ASVs detected during extraction and PCR controls were removed from the dataset. To estimate whether the sequencing depth of the remaining reads was enough to reach sufficient coverage, rarefaction curves were drawn using the *rarecurve* command on package Vegan v 2.6.4. The number of observed ASVs was used as a richness estimate and calculated using the *richness* command in phyloseq to estimate alpha diversity.

### Metabolite identification

An accurate aliquot of the milled grains was placed in a bead beater tube (15 mL, Bertin Technologies, Montingny-le-Bretonneux, France) filled with ceramic balls (zirconium oxide; 6.8 mm), mixed with aqueous EtOH (500 mg/5 mL, 70% EtOH, each) and stored overnight in the freezer at − 20 °C. The Precellys® homogenizer (Bertin Technologies, Montingny-le-Bretonneux, France) was used for sample extraction using the following parameters: 6300 rpm, 3 × 30 s, 15 s pause. After centrifugation (5810R, 4000 rpm, 10 min at 15 °C, Eppendorf, Hamburg, Germany), the supernatant was removed, membrane filtered (0.45 µm Chromafil, Macherey–Nagel, Düren, Germany), and stored in the freezer at − 20 °C until UPLC-MS analysis. Maltose, sucrose, and trehalose were purchased from VWR (Darmstadt, Germany). Asperuloside was purchased from Biomol (Hamburg, Germany). Chromatography solvents, ACN, and methanol for mass spectrometry were purchased from CLN (Niederhummel, Germany) in LC-MS purity. Water as solvent was used after Millipore filtration with an AQUA-Lab – B30 – Integrity system (AQUA-Lab, Ransbach-Baumbach, Germany), and aqueous solvents for chromatography were refreshed after one week. Formic acid as a modifier for chromatography was purchased from Merck (Darmstadt, Germany) in purity > 98%.

#### Ultra performance liquid chromatography

Aliquots (3 *µ*L) of the wheat samples were analyzed in five replicates using UPLC-ESI-TOF MS on a Waters Synapt G2-S HDMS mass spectrometer (Waters, Manchester, UK) coupled to an Acquity UPLC core system (Waters, Milford, MA, USA) equipped with a 2 × 150 mm, 1.7 µm, BEH C18 column (Waters, Manchester) consisting of a binary solvent manager, sample manager and column oven. Operated with a flow rate of 0.4 mL min^−1^ at 50 °C, the following gradient was used for chromatography: starting with a mixture (1/99, v/v) of aqueous HCO_2_H (0.1% in H_2_O) and MeCN (0.1% HCO_2_H) for 0.3 min, the MeCN content was increased to 60% within 3.7 min, to 100% within 6 min, kept constant for 1 min, decreased to 1% within 1 min and finally kept constant for 1.5 min at 1%. The MSe method (centroid) scan time was set to 0.2 s. Analyses were performed with negative ESI in high-resolution mode using the following ion source parameters: capillary voltage − 2.0 kV, sampling cone 50 V, source offset 30 V, source temperature 120 °C, dissolving temperature 450 °C, cone gas flow 2 L h^−1^, nebulizer 6.5 bar and dissolving gas 800 L h^−1^. Data was processed using MassLynx 4.2 SCN 1003 (Waters, Manchester) and the elemental composition tool to determine the accurate mass. All data were lock mass corrected on the pentapeptide leucine enkephaline (Tyr-Gly-Gly-Phe-Leu, *m/z* (mass-to-charge ratio) 554.2615, [M–H]^−^) in a solution (1 ng µL^−1^) of MeCN/0.1% HCO_2_H (1/1, v/v). Scan time for the lock mass was set to 0.3 s, an interval of 15 s, and three scans to average with a mass window of ± 0.3 Da. Calibration of the Synapt G2-S in the range from *m/z* 50 to 1200 was performed using a solution of HCO_2_Na (5 mmol L^−1^) in 2-propanol/H_2_O (9/1, v/v). The UPLC and Synapt G2-S systems were operated with MassLynx™ software (Waters). The collision energy ramp for MS^e^ was set from 20 to 40 eV.

The raw data of all samples and replicates obtained from UPLC-ESI-TOF MS analysis were processed with Progensis QI using the following peak picking conditions: all runs, limits automatic, sensitivity 3, and retention time limits 0.7–11.0 min. Compounds used for principal components analysis (PCA) were filtered using Anova *p*-value ≤ 0.05 and a fold change of ≥ 2. The processed data was exported to EZinfo, where PCA analyzed the matrix using Pareto scaling. Group differences were calculated using orthogonal partial least squares-discriminant analysis (OPLS-DA) highlighted as S-plots.

### Wheat immunoreactive components

#### Wheat protein extraction

The wheat flour proteins were extracted according to a stepwise procedure to obtain the albumin/globulin, gliadin, and glutenin fractions^31^. First, 1 mL of salt solution (0.4 mol L^−1^ NaCl in 0.067 mol L^−1^ Na_2_PO_4_/KH_2_PO_4_ (pH 7.6)) was added to 100 mg of flour. The suspension was mixed for 2 min, stirred for 10 min, and centrifuged (3550 rcf, 25 min, 22 °C). The extraction was repeated, and both supernatants were combined to make up 2 mL (albumins/globulins). Second, 0.5 mL of 60% aqueous ethanol (v + v) was added to the residue, followed by the same mixing, stirring, and centrifugation steps as before. The extraction was repeated twice, and the three supernatants were combined to make up 2 mL (gliadins). Third, 1 mL of glutenin extraction solution (50% (v/v) 2-propanol + 0.1 mol L^−1^ TRIS HCl (pH 7.5) + 2 mol L^−1^ urea + 1% (w/v) dithiothreitol) was added to the residue under argon atmosphere, followed by vortex mixing for 2 min, magnetic stirring for 30 min at 60 °C in a water bath and centrifugation (3550 rcf, 25 min, 22 °C). The extraction was repeated once, and both supernatants were combined and made up to 2 mL (glutenins).

#### Analysis of wheat protein fractions by RP-HPLC

The albumin/globulin, gliadin, and glutenin fractions were analyzed by RP-HPLC with UV detection at 210 nm on a Jasco XLC HPLC (Jasco Deutschland GmbH, Pfungstadt, Germany) using a Dionex Acclaim 300 C18 (3 µm, 2.1 × 150 mm) at 60 °C^32^. Water and acetonitrile containing 0.1% trifluoroacetic acid (TFA) were used as solvents A and B, respectively. The flow rate was 0.2 ml min^−1^ using the following linear gradients: albumins/globulins: 0 min 0% B, 0.5 min 20% B, 5 min 60% B, 5.1–9 min 90% B, 9.1 min 0% B; gliadins and glutenins: 0 min 0% B, 0.5 min 24% B, 15 min 56% B, 15.1–19 min 90% B, 19.2 min 0% B. Injection volumes were 10 µl for albumins/globulins and gliadins and 20 µl for glutenins. For calibration, PWG-gliadin (2.5 mg ml^−1^) was dissolved in 60% aqueous ethanol (v/v) (van Eckert et al. 2006) and analyzed in a range from 11.6 to 46.6 µg per injection. The gluten content is the sum of gliadins and glutenins. The percentages of gluten protein types are based on the gluten content.

## Results

### Grain yield and yield components

**Fig. 1:**
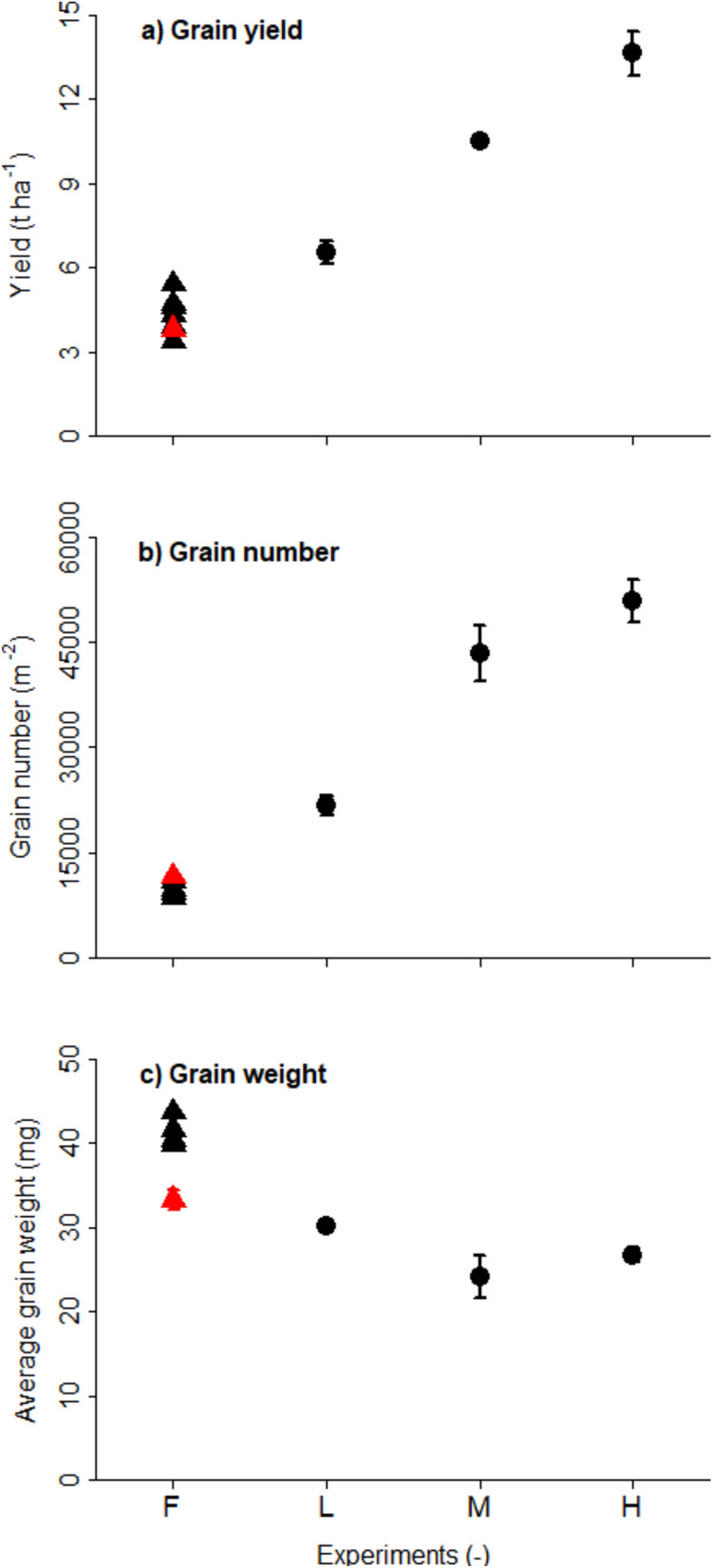
Grain yield and yield components. (**a**) Single-yield harvest in t ha^−1^, (**b**) grain number per m^2^, (**c**) average kernel weight in mg. All measurements at 0% moisture. Field measurements of multiple cultivars (triangles with label F) and indoor measurements with cultivar Apogee (full circles with labels L – low yielding, M – medium yielding, and H – high yielding experiment). The red triangle indicates the cultivar Apogee grown in the field. Vertical bars represent the standard error of biological replications for cultivar Apogee only. There are no replicates for the other individual cultivars grown in the field, and the spread of the cultivars can be considered an indicator of variability in the field.

Like other cultivars, the Apogee cultivar yielded 3.8 t ha^−1^ in the field (Fig. 1). No significant differences in the yields were observed in the other cultivars. In the indoor experiment, where only cultivar Apogee was grown, it yielded 6.5 t ha^−1^ per single harvest in the low (L) -yielding experiment, 10.5 t ha^−1^ per single harvest in the medium (M) -yielding experiment and 13.6 t ha^−1^ per single harvest in the high (H) -yielding experiment, corresponding to a commercial yield at 14% moisture of 7.6, 12.2, and 15.8 t ha^−1^ per single harvest, respectively.

The number of grains per square meter in the field ranged between 8512 and 11,547 m^−2^, and cultivar Apogee reached the highest value observed in the field. In the indoor experiment, the number of grains for cultivar Apogee varied from 21,650 m^−2^ in the L experiment to 50,909 m^−2^ in the H experiment.

While the highest average grain weight measured in the field was 44 mg, cultivar Apogee showed the lowest value with 33 mg. It was even lower in the indoor experiments (30, 24, and 27 mg for L, M, and H experiments, respectively).

**Fig. 2:**
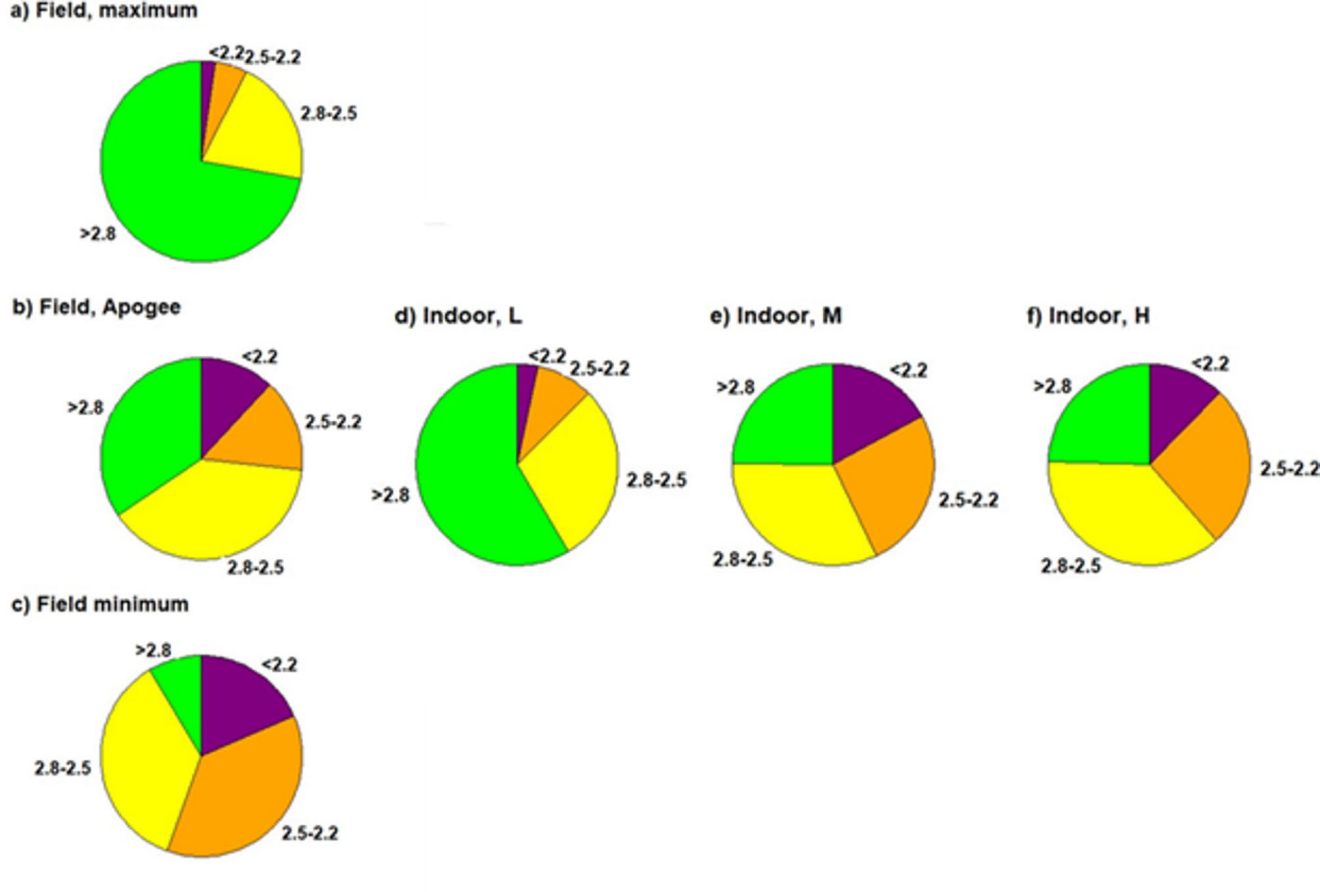
Grain size (diameter in mm) distribution. (**a**) For the cultivar with the largest grain size from the field experiment, (**b**) for the cultivar Apogee grown in the field, (**c**) for the cultivar with the smallest grain size from the field experiment, and cultivar Apogee grown indoors for the (**d**) L- low yielding, (**e**) M – medium yielding and (**f**) H—high yielding experiment.

Figure 2 shows the grain size distribution of the two most different cultivars from the field, cultivar Apogee from the field, and the three indoor treatments. The two fractions with the largest grain size accounted for more than 50% of the grains in all treatments, and Apogee was in the range of the two most different cultivars observed in the field. The share of the two smallest size fractions increased for indoor wheat but remained in the observed grain size distribution range across the cultivars grown in the field.

### Grain quality

**Fig. 3:**
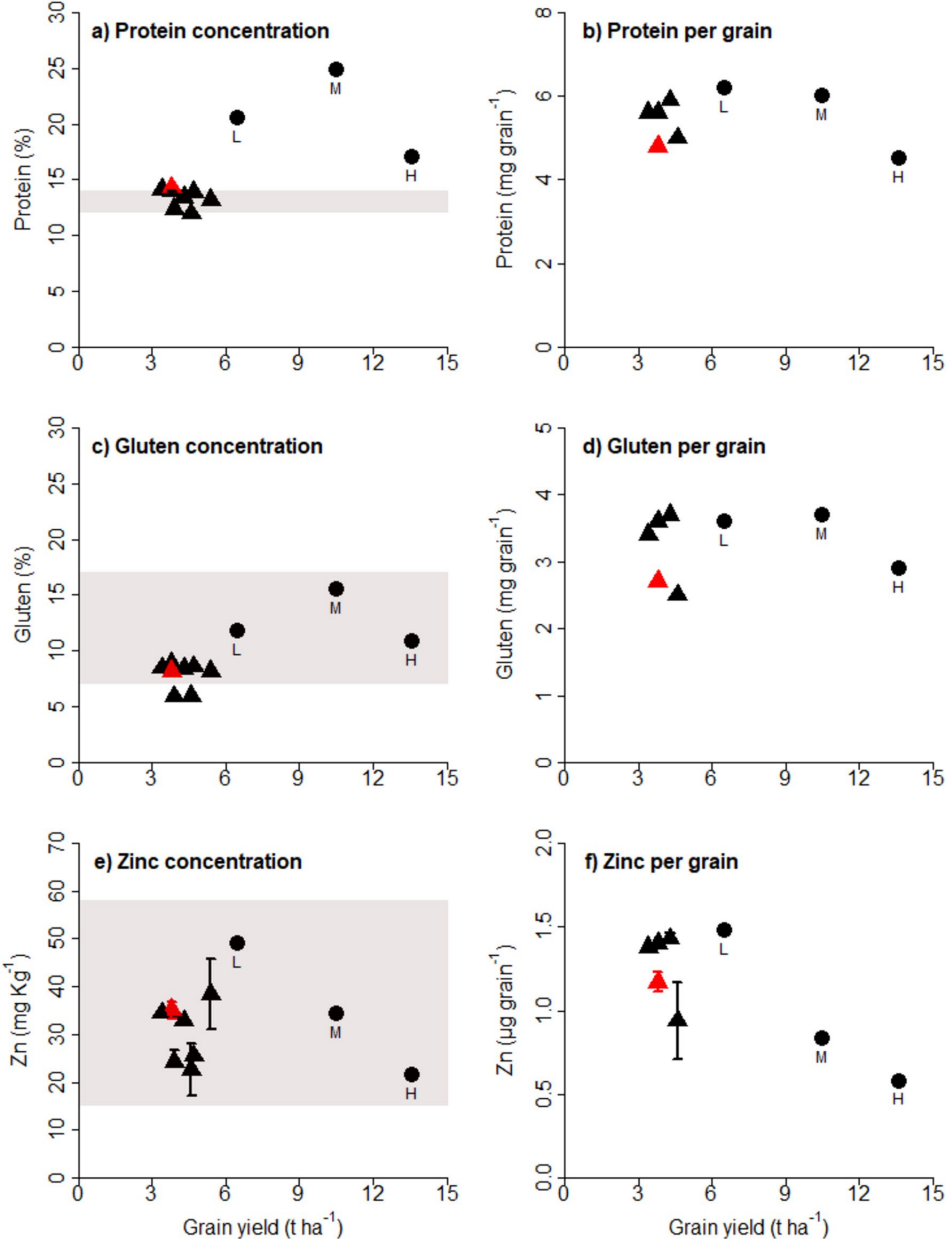
Wheat quality. (**a**, **c**, **e**) Concentration and (**b**, **d**, **f**) weight per average grain for (**a**, **b**) protein in percentage and mg per grain, (**c**, **d**) gluten in percentage and mg per grain, and (**e**, **f**) zinc in mg kg^−1^ and μg per grain for field measurements (triangles) and indoor measurements (full circles). The red triangle indicates the cultivar Apogee grown in the field. (**a**) The grey shaded area indicates the minimum range needed for bread-making quality, but higher protein concentrations have been reported in the field without detrimental effects on bread-making^33^. (**c**, **e**) The grey area indicates the observed range from literature^32,34–36^, as no defined minimum range exists. Means are based on two technical replicates from a mixed sample of each treatment. Vertical bars show standard error. There are no replicates for gluten and for zinc for the indoor M – medium yielding and H – high yielding experiment. For more wheat quality parameters, see Fig. S1–S6.

The grain protein concentration of cultivar Apogee was within the range of the observed protein concentrations of other cultivars grown under field conditions. However, it was significantly higher under indoor conditions, with 24.8% in the M experiment (Fig. 3). The gluten concentration (Fig. 3c) and wet gluten (Supplementary Fig. S1c) followed a similar pattern observed for protein concentration (Fig. 3a). The gluten strength of cultivar Apogee increased with increasing yield potential of indoor environments, whereas in the field environment, all cultivars showed a high to very high gluten index (Supplementary Fig. S1d). Other baking quality parameters, like falling number (Supplementary Fig. S1a), and general quality parameters, like fiber and fat (Supplementary Fig. S2a, c), were affected to a minor extent. Sedimentation significantly increased indoors in the M and H experiments (Supplementary Fig. S1b). Starch (Supplementary Fig. S2e) presented a pattern that was more or less the opposite for protein (Fig. 3a). When expressed in amounts per grain, protein, and gluten (Fig. 3b, d) and fiber, fat, and starch (Supplementary Fig. S2b, d, f) were limited in indoor wheat by grain size, particularly for the M and H experiments, corresponding with the smallest average grain weight (Fig. 1c).

Grain minerals, like Zn concentration, declined for indoor-grown wheat with increasing yields but were within the range observed in the field and of those reported in the literature for wheat (Fig. 3e). Similar to other quality parameters (Fig. 3b, d), when expressed in amounts per grain, Zn (Fig. 3f), for wheat grown indoors, started to indicate grain size as a limitation, particularly for the high-yielding (H) experiment, again, corresponding with the smallest average grain weight (Fig. 1c). Similar patterns were observed for other grain minerals, including P, Mg, K, Cu, and Mn (Supplementary Figs. S3, S4, S5, S6). Ion (Fe) concentration was low in all treatments. Mycotoxins were neither detected in the field nor any of the indoor experiments (data not shown).

### Grain microbiome

**Fig. 4:**
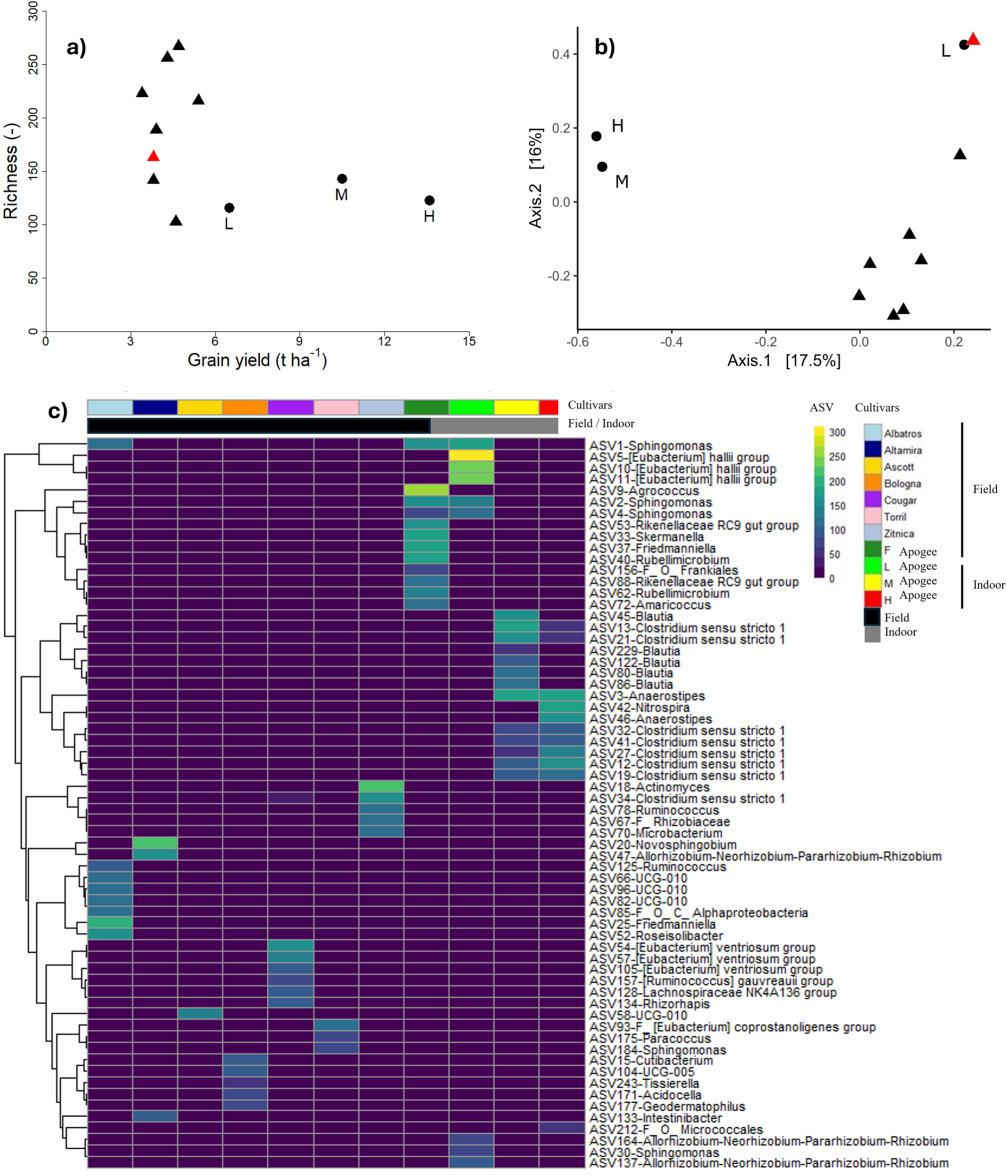
(**a**) Microbiome richness of grain endophytes. The red symbol indicates the cultivar Apogee grown in the field. (**b**) Principal Coordinate Analyses (PCoA) at Bray–Curtis dissimilarity distance display the beta diversity among the different cultivars and the different managing strategies of the Apogee cultivar. The Apogee cultivar coming from the field is shown in red. PERMANOVA analyses showed a significant difference (*p* = 0.002) between the Apogee and the other cultivars samples. (**c**) Heatmap displaying absolute abundance of each Amplicon Sequencing Variant (ASV) assigned at genus level (or last available taxonomic assignment) for seed cultivars in the field, including cultivar Apogee in the field (F) and grown indoors, for the L – low yielding, M – medium yielding and H – high yielding experiment. ASVs with < 50 reads across all samples were removed to improve visualization, and ASVs were assigned only as *Bacteria* and *Proteobacteria* (possible chloroplast contamination). ASVs were clustered using hierarchical clustering based on Euclidean distance and complete linkage. Sample columns were not clustered to preserve the original order.

Bacterial α- diversity, measured as richness inside the wheat grains, differed for the different cultivars in the field trial. For the cultivar Apogee from the field (F), 163 different ASVs were detected, which is in the middle of the other cultivars (Fig. 4a). For the indoor-grown plants, independent from the yield, the bacterial richness in the grains was lower than the field-grown plants for the cultivar Apogee. Interestingly, bacterial richness was highest for the plants that obtained medium yields in the indoor trials. The beta diversity (Fig. 4b) indicates a significant difference (*p* = 0.002) between Apogee and the other cultivars. Figure 4c displays the taxonomical classification of the most abundant ASVs from grains across all samples (> 50 reads) annotated at the genus level. We observed that grains from Apogee cultivars grown in the field remarkably differed from grains of the other cultivars investigated in this study, with a high relative abundance of various genera of *Shingomonas, Agrococcus, Skermanella, Rubelimicrobium* and members of the *Rikenellaceae* and *Frankiales group,* which was not found in grains of the other tested cultivars except for *Shingomonas*, which was also found in grains of the cultivar Albatros. Interestingly, grains from the L treatment had the closest similarity to grains from the outside, with a high dominance of *Shingomonas*. In addition, for those grains, the high relative abundance of bacteria of the Eubacterium “halli” group was remarkable. For grains obtained from plants grown under M and H conditions, bacterial diversity in the grains differed more than those from outside to those from plants grown under L conditions. Most interesting was a shift towards *Clostridium* and *Blautia* in the grains from plants grown under M and H conditions. In addition, for grains from plants grown under H conditions, Nitrospira was high in relative abundance and a member of the family Micrococcus.

### Metabolites

**Fig. 5:**
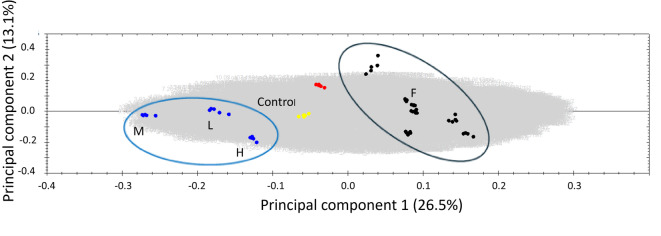
Principal Component Analysis for secondary metabolites in wheat grains from different cultivars (black) grown in the field (F), including cultivar Apogee (red) and the cultivar Apogee grown indoors (blue) for the L- low yielding, M – medium yielding and H – high yielding experiment). Moreover, a control is based on the mix of all samples (yellow). Grey symbols show all mass spectra features used to calculate the PCA. The black ellipsis includes all the samples from the field, and the blue ellipsis is the cultivar Apogee grown indoors. Anova *p*-value <  = 0.05.

To investigate the metabolites composition of the different wheat samples*,* we conducted an untargeted metabolomics approach using UPLC-ESI-TOF MS with simultaneous acquisition of low- and high-collision energy mass spectra (MS^e^), which revealed close clustering of the five technical replicates as well as a clear difference between all samples analyzed (Fig. 5). Employing principal components analysis (PCA), all samples grown in the field (black) and Apogee (red) as a reference could be significantly discriminated against. Apogee grown indoors (Fig. 5, blue) differed mostly along PC1 compared to all other samples. Further, the Apogee indoor samples could be easily separated based on their different yield levels.

In the first analysis, all Apogee grown indoors (L, M, and H) were compared to all field (black + red) samples utilizing Orthogonal Partial Least Squares-Discrimination Analysis (OPLS-DA). To visualize similarities and differences between indoor and field samples, S-plots of data pairs of accurate mass and retention time of each metabolite were calculated (Supplementary Fig. S7). As the y-axis of the S-plot denotes confidence of a metabolite’s contribution to the group difference and the x-axis denotes the contribution of a particular metabolite to the group difference, the S-plot indicates the ions mass-to-charge ratio *m/z* 377.086, 723.3803, 561.3284, 987.6250 as well as 413.1086 showing by far the highest difference for both groups (Supplementary Fig. S7). To highlight the abundance of the most important *m/z* features of the overall wheat samples investigated, their trend plots were summarized in Figs. S7–S27 (Supplementary). The MS features *m/z* 377.086, 723.3803, 561.3284, and 987.6250 were higher in the field samples, and, in comparison, *m/z* 413.1086 was higher in abundance in the indoor samples. Taking into account retention times and accurate *m/z* data, including adduct formation, the following elemental compositions fitting in double bond equivalents could be proposed: for *m/z* 377.086, C_12_H_22_O_11_ a disaccharide; *m/z* 723.3803, C_34_H_60_O_16_ as formic acid adduct of *m/z* 677.3750, C_33_H_58_O_14_, a dipyranosyl-*mono-*glyceride; *m/z* 561.3284, C_28_H_50_O_11_ as formic acid adduct of *m/z* 515.3235, C_27_H_48_O_9,_ a pyranosyl-*mono-*glyceride; *m/z* 987.6250, C_52_H_92_O_17_ as formic acid adduct of *m/z* 941.6208, C_51_H_90_O_15_, a dipyranosyl-*di-*glyceride; as well as *m/z* 413.1086 C_18_H_22_O_11_ an iridoid glycoside (Supplementary Figs. S7–S27).

### Wheat immunoreactive components

**Fig. 6:**
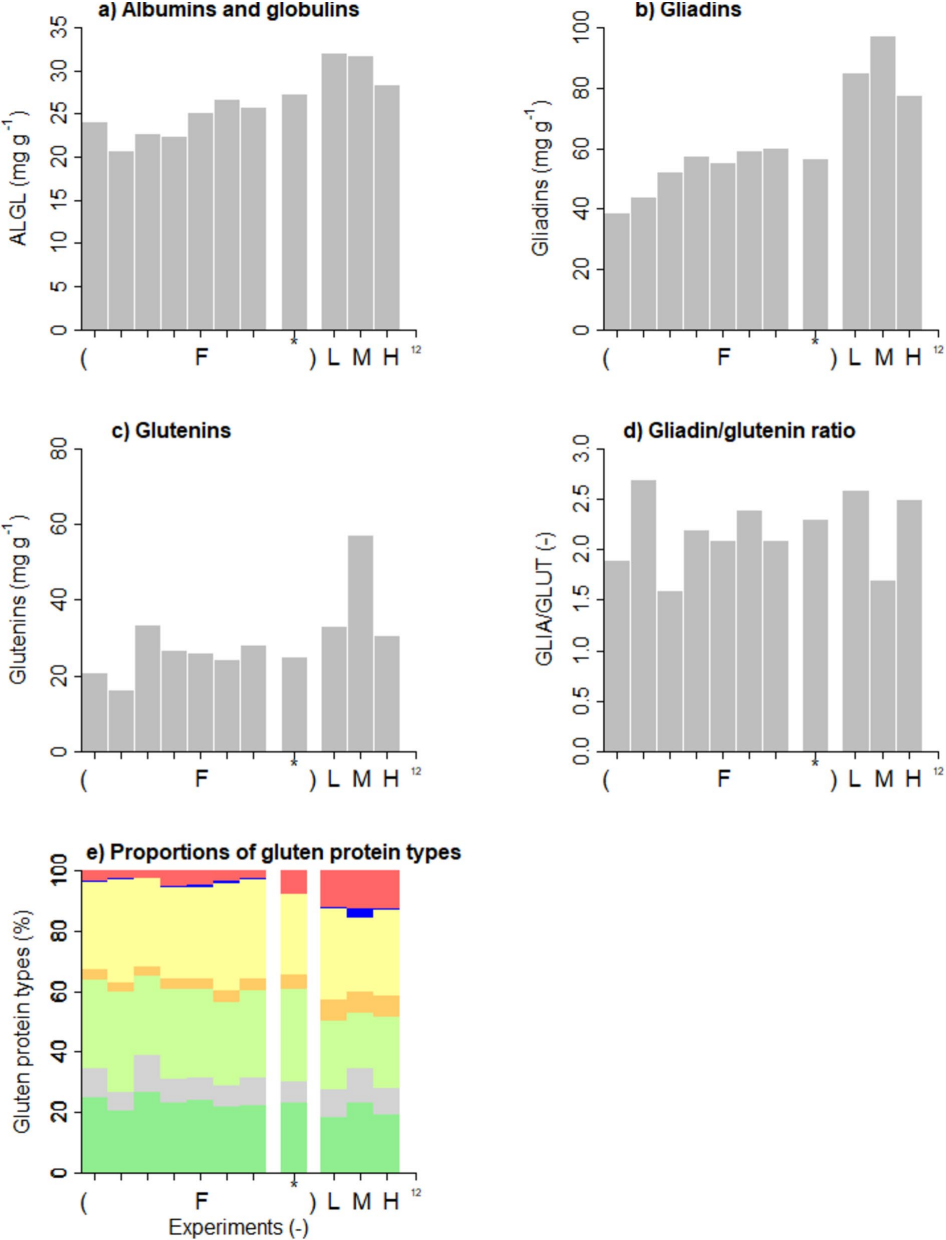
Indicators for bread-making quality and immunoreactive potential for individuals with wheat-related disorders. (**a**) Albumins and globulins in mg g^−1^, (**b**) Gliadin content in mg g^−1^, (**c**) glutenin content in mg g^−1^, (**d**) Gliadin/glutenin ratio and (**e**) percentage proportions of gluten protein types including ω5-gliadins (red), ωb-gliadins (blue), α-gliadins (yellow), ω1,2-gliadins (orange), γ-gliadins (light green), HMW-GS (grey) and LMW-GS (green) relative to total gluten in whole grain flour for cultivars in the field (F), including cultivar Apogee in the field (*), and cultivar Apogee grown indoors, for the L – low yielding, M – medium yielding and H – high yielding experiment.

The content of albumins and globulins was 20.7–27.4 mg g^−1^ of flour for cultivars grown in the field, including Apogee with 27.4 mg g^−1^, whereas that for Apogee grown indoors was slightly higher with 28.5–32.1 mg g^−1^ (Fig. 6a). The cultivars grown in the field had a gliadin content of 38.6–60.3 mg g^−1^ and a glutenin content of 16.4–33.5 mg g^−1^, including Apogee with 56.8 mg g^−1^of gliadins and 25.0 mg g^−1^ of glutenins (Fig. 6b, c). When Apogee was grown indoors, the gliadin content was higher with 77.8–97.5 mg g^−1^, and the glutenin content was comparable or higher with 30.8–57.4 mg g^−1^. Sample M grown for medium yield stood out due to an exceptionally high gliadin and glutenin content. The gliadin/glutenin ratio was between 1.6 and 2.7 for cultivars grown in the field (Fig. 6d). Apogee grown indoors had gliadin/glutenin ratios of 2.6 (L), 1.7 (M), and 2.5 (H), also in the same range, mainly because both the gliadin and the glutenin content tended to be higher compared to the field samples. To get more insights into possible differences in gluten protein composition independent of variable total protein and gluten content, the proportions of ω5-, ωb-, α-, ω1,2- and γ-gliadins as well as HMW-GS and LMW-GS were expressed based on total gluten content (Fig. 6e). The mean proportions of the eight cultivars grown in the field were 4.0% of ω5-gliadins, 0.5% of ωb-gliadins, 30.8% of α-gliadins, 3.8% of ω1,2-gliadins, 29.2% of γ-gliadins, 8.3% of HMW-GS and 23.3% of LMW-GS. The proportions of most gluten protein types for cultivar Apogee grown in the field lay within the range of all eight cultivars, except ω5-gliadins (7.7%) and ω1,2-gliadins (5.0), were higher compared to the other seven cultivars. In contrast, α-gliadins (26.4%) were lower. Considering the mean of the three Apogee samples grown indoors, the gluten protein composition was 12.4% of ω5-gliadins, 1.2% of ωb-gliadins, 27.8% of α-gliadins, 7.1% of ω1,2-gliadins, 21.6% of γ-gliadins, 9.7% of HMW-GS and 20.2% of LMW-GS. In comparison to the mean of the eight cultivars grown in the field, the proportions of ω5-gliadins, ωb-gliadins, ω1,2-gliadins, and HMW-GS were thus higher, and those of α-gliadins, γ-gliadins, and LMW-GS lower in the indoor samples. As already seen in the content of gliadins and glutenins, treatment M showed the most pronounced difference in gluten protein composition compared to treatment L and H. The most remarkable difference was the increase in ω-gliadins in the indoor Apogee treatments compared to the field.

## Discussion

### Grain yield and yield components

The higher indoor grain yield can be attributed to the higher grain number despite the decrease in grain weight. This confirms that, similar to field conditions, grain yield variations in wheat are more closely associated with grain number variations than grain weight^37^. Grain numbers per square meter, determined by the number of ears per square meter and grains per ear, were higher under indoor conditions than in the field due to the increased plant and ear density, which resulted in more ears and grains per square meter. In addition, indoor grain yields were favoured by high light input, favourable temperature, and constant water and nutrient supply. The increase in the two smallest size fractions for indoor wheat could have partly contributed to the lower average grain weight and is consistent with the observed rise in the proportional contribution of distal grains, which are relatively light, as grain numbers increase^38^. Given the low average grain weight attained indoors, an increase in grain yield indoors is expected through an increase in grain weight. It has been demonstrated by Bustos et al.^39^ that even in the field and with the current atmospheric CO₂ concentration, wheat can combine a high grain number (39,000 grains m^−2^, which is about a quarter less than in the indoor high-yielding H experiment) and a high grain weight (43 mg compared to 27 mg in the H treatment). This suggests a further potential to increase the grain weight under indoor conditions by e.g. lengthening the duration of grain filling through a lower temperature after anthesis with a potential achievable yield of 21.9 t ha^−1^ (50,909 grains m⁻^2^ from the indoor high-yielding (H) experiment multiplied with 43 mg average grain size reported by Bustos et al.^39^), still below the estimated potential indoor wheat yield suggested by Asseng et al.^4^. However, this would only be possible with simultaneously increasing the straw biomass to stay within a realistic harvest index^40^. The primary drawback of such an option would be a longer growing cycle if the earlier growth stages cannot be accelerated with higher temperatures and fewer harvests per year. Another approach to increasing grain weight could be to promote a higher number of endosperm cells, which influence the grain filling rate and, ultimately, the final grain weight^41,42^. The number of endosperm cells is determined just after anthesis; therefore, a short period of low temperature would be necessary to lengthen the period of endosperm cell formation and hence to increase their number and, in turn, enhance both the rate of grain filling and the final average grain weight.

### Grain quality

Protein is the primary determinant of grain prices and processing quality^10^. For indoor wheat, protein concentration increased due to the unrestricted water supply and nutrients in hydroponics. Protein in the medium-yielding M experiment exceeded the values observed in the literature by nearly three percentage points^33^, suggesting that the decline in protein concentration observed with increasing grain yield in field experiments may depend on limited nitrogen supply^8,10^. Increasing N-supply, thus resulting in high yield and very high protein contents, could be achieved in indoor farming conditions.

High protein contents are desirable for mills and are mixed with suboptimal protein contents to achieve flour with protein contents of baking quality. However, the increase in yield in indoor wheat was accompanied by a decrease in grain weight and size, which might limit the use for bread making, as smaller grain size leads to lower flour yields and higher bran content. This also suggests suboptimal growing conditions during grain filling, highlighting the need for further research on separately optimizing growth conditions within different growth phases. For wheat processing, grain size and grain weight are critical parameters^43^. At the same time, starch was also affected by the decrease in grain weight. Enhancing grain filling duration or grain filling rate may promote more active starch synthesis after anthesis and accumulation into the grains, leading to more extensive and heavier grains^44^. Since starch synthesis in crops predominantly occurs at night^45^, extending dark cycles during grain filling might increase the average grain size.

Although the increase in some macro-elements such as P and K may be related to an increase in their supply^46^, similarly to the starch concentration, the decline of micro-nutrients such as Zn and Mn might be explained by the capacity of the crop to accumulate them during the grain filling^8^. Other micro-element concentrations, such as Fe, were low in all growing environments, suggesting a possible impact by the cultivars. In particular, it has been reported that short-strawed varieties may be less efficient in translocating minerals to the grains than photosynthates^33^.

The high grain protein concentration observed indoors could also serve as a valuable raw material in the processing industry, helping to balance low protein batches or contributing to specialized dough properties in baking goods. These findings highlight the potential of indoor wheat farming to optimize wheat quality based on specific industrial requirements. However, while the measured parameters suggest a promising potential for the indoor-grown wheat bread-making, further studies should include empirical baking tests to validate the functional baking performance.

### Grain microbiome

The plant-associated microbiome provides essential functions that determine plant fitness and quality^17^. For indoor-grown plants, microbial functions linked to the biocontrol of plant pathogens and plant growth promotion might be essential triggers for plant health. In contrast to outdoor plants, which recruit major parts of their microbiome from the environment (mainly soil)^47^, indoor-grown wheat plants solely depend on the vertical transmission of microbes via the grain-associated microbiome from generation to generation^48^. Thus, the consequences of indoor cultivation of plants for the composition of the seed microbiome are essential to forecast plant health.

This study shows that indoor cultivation of plants, from seed lines maintained indoors for several generations, results in a slight decrease in the overall diversity of bacteria in the grains but significant changes in the dominant taxa, mainly if plants were grown under medium and high-yielding conditions. Grains from plants grown under low-yielding conditions were more similar to those grown under field conditions and dominated by *Shingomonas*, which almost disappeared with higher indoor yields. The genus *Sphingomonas* consists of Gram-negative, aerobic bacteria commonly found in soil, water, and plant-associated environments. These bacteria are known for their ability to degrade complex organic compounds, promote plant growth, and enhance plant resilience against biotic and abiotic stresses^49^. One of the key mechanisms through which *Sphingomonas* contributes to plant growth promotion is its role in phosphate solubilization. Many species produce organic acids that convert insoluble phosphates into bioavailable forms, improving nutrient uptake by plants^50^. Additionally, *Sphingomonas* strains have been shown to produce indole-3-acetic acid (IAA), a plant hormone that stimulates root elongation and lateral root formation, enhancing overall plant development^51^. In addition, *Sphingomonas* strains are well described as heaving biocontrol activity^52^. Certain strains exhibit antagonistic properties against plant pathogens by producing antimicrobial compounds or inducing systemic resistance in host plants. For example, *Sphingomonas sp.* has been reported to suppress fungal infections in crops like wheat and rice by outcompeting harmful microbes and modulating plant defense responses^53^. However, fungal infections might be less of an issue in pest-free indoor cultivation. Finally, *Sphingomonas* is crucial in mitigating abiotic stress, which is also less of a problem in well-controlled indoor growing conditions^54^.

A loss of bacteria of these genera in the grains might also affect plant health under indoor conditions. The substantial increase in relative abundance of *Clostridium* and *Blautia* in grains from the high- and medium-yielding conditions does not compensate for the losses of *Sphingomonas*, as the traits provided by those bacterial genera differ. The production of plant growth regulators like indole-3-acetic acid (IAA) has been described only for some Clostridium species ^55^.

The presence of plant growth-promoting bacteria, such as the *Sphingomonas* and *Agroccocus* in the Apogee cultivar, has already been demonstrated to improve plant growth, yield, and nutrient content when inoculated in strawberry^56^ and soy^57^. Those studies suggest that the several beneficial effects of those inoculated bacteria, such as auxin and cytokinin production and N_2_-fixation, can stimulate plant growth and stabilize plant growth during stressful conditions^58^, therefore increasing plant yield and overall nutrient content. Here, we demonstrated that those taxa are naturally enriched in the Apogee cultivar, compared to the other investigated cultivars, which can help to explain the increasing yield.

### Metabolite identification

Using the strategy described for the identification of digalacto-mono/di-acylglycerols in literature^59^ and the observed accurate mass fragment data (Supplementary Figs. S7–S27) as well as the knowledge about mono- and digalacto-mono/di-acylglycerols published in the literature in wheat^60,61^ the following marker compounds revealing significant higher intensities in the field samples were identified as:

*m/z* 723.3803 (*m/z* 677.3750) as 1-linoleoyl-3-*O*-(*β*-D-digalactopyranosyl)-*sn-*glycerol (DGMG-L); *m/z* 561.3284 (*m/z* 515.3235) as 1-linoleoyl-3-*O*-(*β*-D-galactopyranosyl)-*sn-*glycerol (MGMG-L); *m/z* 987.6250 (*m/z* 941.6208) as 1-oleoyl-2-linoleoyl-3-*O*-(*β*-D-digalactopyranosyl)-*sn-*glycerol (DGDG-OL) or the constitutional isomer 1-linoleoyl-2-oleoyl-3-*O*-(*β*-D-digalactopyranosyl)-*sn-*glycerol (DGDG-LO). Further, discrimination between the disaccharides could be achieved by injecting the references trehalose, sucrose as well as maltose, which revealed a slightly earlier elution for sucrose and maltose in the chromatogram, as well as the fact that trehalose additionally ionizes as a chloride ion *m/z* 377^62^ (M–H: *m/z* 341), underlies the identification of *m/z* 377.086 as trehalose and a marker compound for the field samples. In contrast, by in-depth analysis of the fragments of *m/z* 413.1086 and published compounds in wheat^62^ and purchasing the reference compound, the maker for indoor samples asperuloside, an iridoid glycoside, could be unambiguously identified.

Further comparisons, e.g., all Apogee grown indoor versus only Apogee grown in the field (red) samples by OPLS-DA (Supplementary Fig. S24), as well as only Apogee indoor (medium-yielding) versus field grown varieties (Supplementary Fig. S25), medium-yielding Apogee indoor versus low-yielding Apogee indoor (Supplementary Fig. S26) and high-yielding Apogee indoor versus low-yielding Apogee indoor (Supplementary Fig. S27) revealed one after another with the different impact the above-described maker compounds.

This is the first HR-ESI-LC–MS-based metabolomics study, which compared soilless/artificial light indoors versus a field-based grown crop, here with wheat. The identified galactolipids in indoor wheat exhibit excellent baking performance, but how the differences in their abundances (Supplementary Figs. S8-14 and S18-20) affect this baking quality remains an open question. The galactolipids will need to be quantified and baking experiments and rheological investigations should be performed^63^ to target their different amounts. However, other ingredients, like lipids, starch, minerals, and proteins, especially gluten, as discussed above under grain quality, are also important for bread-making quality and warrant further investigations.

For asperuloside, which was higher indoors than field-grown wheat, anticancer or attenuation of cadmium-induced toxicity by inhibiting oxidative stress, inflammation, fibrosis, and apoptosis in rats have been reported^64,65^.

### Wheat immunoreactive components

The overall content of albumins and globulins of flour for cultivars grown in the field, including cultivar Apogee and for Apogee indoors, was characteristic for whole grain common wheat flours and comparable to reports elsewhere^66^. The range of gliadin and glutenin contents of the cultivars grown in the field, including Apogee, was similar to other field-based reports^32,66^. When Apogee was grown indoors, the gliadin and glutenin contents were higher. The medium-yielding experiment stood out due to an exceptionally high gliadin and glutenin content outside the typical range^67^. The gliadin/glutenin ratio for cultivars grown in the field and cultivar Apogee grown indoors was similar under both growing conditions and in accordance with previous studies^32,66^, indicating no change in the estimated baking properties of indoor-grown wheat. The mean proportions of ω5-, ωb-, α-, ω1,2- and γ-gliadins as well as HMW-GS and LMW-GS for the eight cultivars grown in the field were in line with earlier studies^32,67^. The most remarkable difference was the increase in ω-gliadins in the indoor Apogee grains. For bread-making quality, the S-poor ω-gliadins are assumed to play a minor role because they cannot form intermolecular disulfide bonds within the gluten network due to their lack of cysteine (ω5/ω1,2) or the presence of only one cysteine residue (ωb)^68^. However, there is evidence of non-covalent interactions between ω-gliadins and polymeric glutenins^69^, which is why an increase of more than twofold in the proportion of ω-gliadins at the expense of other gluten protein types is likely to have a positive impact on bread-making quality. Further, the proportion of HMW-GS, the primary type of gluten protein associated with bread-making quality^70^, increased in indoor grains, which is why the dough and bread-making properties of indoor samples will need to be assessed directly in future experiments. Concerning wheat-related disorders, the relative increase of ω5-gliadins in indoor samples is detrimental because they are the primary triggers of wheat-dependent, exercise-induced anaphylaxis, a life-threatening food allergy^71^. For celiac disease, the relative decrease of α-gliadins and γ-gliadins in indoor grains may be beneficial because these two types contain the highest numbers of known celiac disease-relevant epitopes recognized by CD4^+^ T cells^72^. However, the overall increase in gluten content in indoor grains is likely to counter-balance any relative decrease in certain gluten protein types because all gluten proteins have known epitopes. Detailed proteomics experiments focusing on peptides with known epitopes similar to Norwig et al.^73^ would be needed to make a more precise assessment of the immunogenic potential of indoor-grown wheat but were outside of the current study. Since starch is the grain’s primary component, increasing grain size and starch content can influence the protein levels^74^. This may result in slight reductions in gluten proteins and their proportions, which can positively affect grain yield, milling quality, and the immunoreactive potential of gluten proteins. The advantage of growing crops indoors is that all growth conditions can be controlled precisely, ideally offering the opportunity to tailor the gluten protein composition in a way that is beneficial for both bread-making quality and reduced immunoreactive potential.

## Conclusion

Indoor-grown wheat without soil and with artificial light can achieve high yields with better indicators of bread-making quality compared to modern spring wheat cultivars growing in a field. The controlled indoor conditions had a minor effect on the microbiome diversity inside the grain. At the same time, there were differences in the presence of secondary metabolites and slight changes in protein composition, enhancing bread-making quality. Immunoreactive proteins were present regardless of the growing environment. However, the change in the amount and ratio of gluten proteins may impact the immunoreactive potential in the context of wheat allergy and celiac disease. Growing wheat indoors in a vertical farm has the potential to significantly contribute to food supply and nutrition worldwide. However, research is still needed to understand how to control food quality and health-related compounds while simultaneously increasing grain yields in indoor growing conditions. Finally, the high energy demand for light remains the main barrier to the commercialisation of vertical wheat farming. However, advances in the vertical farming technology and the adoption of renewable energy sources will reduce overall costs and profitability issues, positioning vertical farming as a possible strategy to support food security.

## Supplementary Information

Supplementary Information.

## Data Availability

The datasets generated and analyzed during the current study are available from the corresponding author on request. Raw sequencing files were uploaded to the NCBI SRA database under the BioProject number PRJNA1218620, BioSample SAMN46786272. (https://www.ncbi.nlm.nih.gov/bioproject/1218620).

## References

[1] Ray, D. K., Ramankutty, N., Mueller, N. D., West, P. C. & Foley, J. A. Recent patterns of crop yield growth and stagnation. *Nat. Commun.* **3**, 1293 (2012). 23250423 10.1038/ncomms2296

[2] Ray, D. K., Mueller, N. D., West, P. C. & Foley, J. A. Yield trends are insufficient to double global crop production by 2050. *PLoS ONE* **8**, e66428 (2013). 23840465 10.1371/journal.pone.0066428 PMC3686737

[3] Keating, B. A., Herrero, M., Carberry, P. S., Gardner, J. & Cole, M. B. Food wedges: Framing the global food demand and supply challenge towards 2050. *Glob. Food Sec.* **3**, 125–132 (2014).

[4] Asseng, S. et al. Wheat yield potential in controlled-environment vertical farms. *Proc. Natl. Acad. Sci.* **117**, 19131–19135 (2020). 32719119 10.1073/pnas.2002655117 PMC7430987

[5] Peltonen-Sainio, P., Salo, T., Jauhiainen, L., Lehtonen, H. & Sieviläinen, E. Static yields and quality issues: Is the agri-environment program the primary driver?. *Ambio* **44**, 544–556 (2015). 25663562 10.1007/s13280-015-0637-9 PMC4552720

[6] Myers, S. S. et al. Increasing CO_2_ threatens human nutrition. *Nature* **510**, 139–142 (2014). 24805231 10.1038/nature13179 PMC4810679

[7] Wieser, H., Manderscheid, R., Erbs, M. & Weigel, H.-J. Effects of elevated atmospheric CO_2_ concentrations on the quantitative protein composition of wheat grain. *J. Agric. Food Chem.* **56**, 6531–6535 (2008). 18598044 10.1021/jf8008603

[8] Broberg, M. C., Högy, P. & Pleijel, H. CO_2_-induced changes in wheat grain composition: meta-analysis and response functions. *Agronomy* **7**, 32 (2017).

[9] Pleijel, H., Broberg, M. C., Högy, P. & Uddling, J. Nitrogen application is required to realize wheat yield stimulation by elevated CO_2_ but will not remove the CO_2_-induced reduction in grain protein concentration. *Glob. Change Biol.* **25**, 1868–1876 (2019). 10.1111/gcb.14586 30737900

[10] Blumenthal, C., Rawson, H. & McKenzie, E. Changes in wheat grain quality due to doubling the level of atmospheric CO(2). *Cereal Chem.* **73**, 762–766 (1996).

[11] Rogers, G. et al. The influence of atmospheric CO_2_ concentration on the protein, starch and mixing properties of wheat flour. *Funct. Plant Biol.* **25**, 387–393 (1998).

[12] Högy, P. et al. Effects of elevated CO_2_ on grain yield and quality of wheat: results from a 3-year free-air CO_2_ enrichment experiment. *Plant Biol.* **11**, 60–69 (2009). 19778369 10.1111/j.1438-8677.2009.00230.x

[13] Dreccer, M. F. et al. Wheat yield potential can be maximized by increasing red to far-red light conditions at critical developmental stages. *Plant, Cell Environ.* **45**, 2652–2670 (2022). 35815553 10.1111/pce.14390

[14] Monostori, I. et al. LED lighting–modification of growth, metabolism, yield and flour composition in wheat by spectral quality and intensity. *Front. Plant Sci.* **9**, 605 (2018). 29780400 10.3389/fpls.2018.00605 PMC5945875

[15] Clauw, H. et al. The impact of a six-hour light-dark cycle on wheat ear emergence, grain yield, and flour quality in future plant-growing systems. *Foods* **13**, 750 (2024). 38472863 10.3390/foods13050750 PMC10931310

[16] Wieser, H., Koehler, P. & Scherf, K. A. The two faces of wheat. *Front. Nutr.* **7**, 517313 (2020). 33195360 10.3389/fnut.2020.517313 PMC7609444

[17] Berg, G., Grube, M., Schloter, M. & Smalla, K. Unraveling the plant microbiome: looking back and future perspectives. *Front. Microbiol.* **5**, 148 (2014). 24926286 10.3389/fmicb.2014.00148 PMC4045152

[18] Welch, B. L. The generalization of ‘STUDENT’S’problem when several different population varlances are involved. *Biometrika* **34**, 28–35 (1947). 20287819 10.1093/biomet/34.1-2.28

[19] Bugbee, B. & Koerner, G. Yield comparisons and unique characteristics of the dwarf wheat cultivar ‘USU-Apogee’. *Adv. Space Res.* **20**, 1891–1894 (1997). 11542565 10.1016/s0273-1177(97)00856-9

[20] https://gml.noaa.gov/ccgg/trends/. (2022).

[21] https://gml.noaa.gov/ccgg/trends/. (2023).

[22] Jákli, B. et al. Regionalized dynamic climate series for ecological climate impact research in modern controlled environment facilities. *Ecol. Evol.* **11**, 17364–17380 (2021). 34938514 10.1002/ece3.8371 PMC8668799

[23] Lueders, T., Manefield, M. & Friedrich, M. W. Enhanced sensitivity of DNA-and rRNA-based stable isotope probing by fractionation and quantitative analysis of isopycnic centrifugation gradients. *Environ. Microbiol.* **6**, 73–78 (2004). 14686943 10.1046/j.1462-2920.2003.00536.x

[24] Dorn-In, S., Bassitta, R., Schwaiger, K., Bauer, J. & Hölzel, C. S. Specific amplification of bacterial DNA by optimized so-called universal bacterial primers in samples rich of plant DNA. *J. Microbiol. Methods* **113**, 50–56 (2015). 25863142 10.1016/j.mimet.2015.04.001

[25] Martin, M. Cutadapt removes adapter sequences from high-throughput sequencing reads. *EMBnet. J.* **17**, 10–12 (2011).

[26] Wingett, S. W. & Andrews, S. FastQ Screen: A tool for multi-genome mapping and quality control. *F1000Research* **7**, 1338 (2018). 30254741 10.12688/f1000research.15931.1 PMC6124377

[27] Callahan, B. J. et al. DADA2: High-resolution sample inference from Illumina amplicon data. *Nat. Methods* **13**, 581–583 (2016). 27214047 10.1038/nmeth.3869 PMC4927377

[28] Quast, C. et al. The SILVA ribosomal RNA gene database project: Improved data processing and web-based tools. *Nucleic Acids Res.* **41**, D590–D596 (2012). 23193283 10.1093/nar/gks1219 PMC3531112

[29] McMurdie, P. J. & Holmes, S. phyloseq: An R package for reproducible interactive analysis and graphics of microbiome census data. *PLoS ONE* **8**, e61217 (2013). 23630581 10.1371/journal.pone.0061217 PMC3632530

[30] Oksanen, J. et al. vegan community ecology package version 2.6-2 April 2022. The Comprehensive R Archive Network. Available online: http://cran.r-project.org (accessed on 15 August 2022) (2022).

[31] Wieser, H., Antes, S. & Seilmeier, W. Quantitative determination of gluten protein types in wheat flour by reversed-phase high-performance liquid chromatography. *Cereal Chem.* **75**, 644–650 (1998).

[32] Schuster, C., Huen, J. & Scherf, K. A. Comprehensive study on gluten composition and baking quality of winter wheat. *Cereal Chem.* **100**, 142–155 (2023).

[33] Shewry, P. R. Wheat. *J. Exp. Bot.* **60**, 1537–1553. 10.1093/jxb/erp058 (2009). 19386614 10.1093/jxb/erp058

[34] Bodor, K., Szilágyi, J., Salamon, B., Szakács, O. & Bodor, Z. Physical–chemical analysis of different types of flours available in the Romanian market. *Sci. Rep.* **14**, 881 (2024). 38195806 10.1038/s41598-023-49535-x PMC10776669

[35] Piironen, V., Lampi, A.-M., Ekholm, P., Salmenkallio-Marttila, M. & Liukkonen, K.-H. in Wheat: chemistry and technology 179–222 (AACC International, 2009).

[36] Dendy, D. A. & Dobraszczyk, B. J. *Cereals and cereal products: Technology and chemistry* (Aspen Publishers, 2001).

[37] Fischer, R. Wheat physiology: a review of recent developments. *Crop Pasture Sci.* **62**, 95–114 (2011).

[38] Acreche, M. M. & Slafer, G. A. Grain weight response to increases in number of grains in wheat in a Mediterranean area. *Field Crop Res* **98**, 52–59 (2006).

[39] Bustos, D. V., Hasan, A. K., Reynolds, M. P. & Calderini, D. F. Combining high grain number and weight through a DH-population to improve grain yield potential of wheat in high-yielding environments. *Field Crop Res* **145**, 106–115 (2013).

[40] Foulkes, M. J. et al. Raising yield potential of wheat III. Optimizing partitioning to grain while maintaining lodging resistance. *J. Exp. Botany* **62**, 469–486 (2011). 20952627 10.1093/jxb/erq300

[41] Brocklehurst, P. Factors controlling grain weight in wheat. *Nature* **266**, 348–349 (1977).

[42] Singh, B. & Jenner, C. Association between concentration of organic nutrients in the grain, endosperm cell number and grain dry weight within the ear of wheat. *Funct. Plant Biol.* **9**, 83–95 (1982).

[43] Marshall, D., Mares, D., Moss, H. & Ellison, F. Effects of grain shape and size on milling yields in wheat II. Experimental studies. *Austral. J. Agricult. Res.* **37**, 331–342 (1986).

[44] Pan, J., Zhu, Y. & Cao, W. Modeling plant carbon flow and grain starch accumulation in wheat. *Field Crop Res.* **101**, 276–284 (2007).

[45] Streb, S. & Zeeman, S. C. Starch metabolism in Arabidopsis. *The arabidopsis book/American society of plant biologists* **10**, e0160 (2012). 10.1199/tab.0160 PMC3527087 23393426

[46] Uthayakumaran, S. & Wrigley, C. *Cereal Grains* 91–134 (Elsevier, 2017).

[47] Berendsen, R. L., Pieterse, C. M. & Bakker, P. A. The rhizosphere microbiome and plant health. *Trends Plant Sci.* **17**, 478–486 (2012). 22564542 10.1016/j.tplants.2012.04.001

[48] War, A. F., Bashir, I., Reshi, Z. A., Kardol, P. & Rashid, I. Insights into the seed microbiome and its ecological significance in plant life. *Microbiol. Res.* **269**, 127318 (2023). 36753851 10.1016/j.micres.2023.127318

[49] Kolvenbach, B. & Corvini, P.-X. The degradation of alkylphenols by Sphingomonas sp. strain TTNP3–a review on seven years of research. *New Biotechnol.* **30**, 88–95 (2012). 10.1016/j.nbt.2012.07.008 22842087

[50] Asaf, S., Numan, M., Khan, A. L. & Al-Harrasi, A. Sphingomonas: from diversity and genomics to functional role in environmental remediation and plant growth. *Crit. Rev. Biotechnol.* **40**, 138–152 (2020). 31906737 10.1080/07388551.2019.1709793

[51] Khan, A. L. et al. Bacterial endophyte Sphingomonas sp. LK11 produces gibberellins and IAA and promotes tomato plant growth. *J Microbiol* **52**, 689–695 (2014). 24994010 10.1007/s12275-014-4002-7

[52] Yang, Z. et al. Biocontrol agents modulate phyllosphere microbiota interactions against pathogen Pseudomonas syringae. *Environ. Sci. Ecotechnol.* **21**, 100431 (2024). 38883559 10.1016/j.ese.2024.100431 PMC11177076

[53] Lombardino, J. et al. Genomic characterization of potential plant growth-promoting features of Sphingomonas strains isolated from the International Space Station. *Microbiol. Spectrum* **10**, e01994–e019921 (2022). 10.1128/spectrum.01994-21 PMC8754149 35019675

[54] Ali, U., Li, H., Wang, X. & Guo, L. Emerging roles of sphingolipid signaling in plant response to biotic and abiotic stresses. *Mol. Plant* **11**, 1328–1343 (2018). 30336328 10.1016/j.molp.2018.10.001

[55] Whitehead, T. R., Price, N. P., Drake, H. L. & Cotta, M. A. Catabolic pathway for the production of skatole and indoleacetic acid by the acetogen Clostridium drakei, Clostridium scatologenes, and swine manure. *Appl. Environ. Microbiol.* **74**, 1950–1953 (2008). 18223109 10.1128/AEM.02458-07 PMC2268313

[56] Esitken, A. et al. Effects of plant growth promoting bacteria (PGPB) on yield, growth and nutrient contents of organically grown strawberry. *Sci. Hortic.* **124**, 62–66 (2010).

[57] Moretti, L. G. et al. Effects of growth-promoting bacteria on soybean root activity, plant development, and yield. *Agron. J.* **112**, 418–428 (2020).

[58] Azarbad, H., Bainard, L. D., Agoussar, A., Tremblay, J. & Yergeau, E. The response of wheat and its microbiome to contemporary and historical water stress in a field experiment. *ISME Commun.* **2**, 62 (2022). 37938737 10.1038/s43705-022-00151-2 PMC9723694

[59] Stark, T. D., Weiss, P., Friedrich, L. & Hofmann, T. The wheat species profiling by non-targeted UPLC–ESI–TOF-MS analysis. *Eur. Food Res. Technol.* **246**, 1617–1626 (2020).

[60] Prieto, J., Ebri, A. & Collar, C. Composition and distribution of individual molecular species of major glycolipids in wheat flour. *J. Am. Oil. Chem. Soc.* **69**, 1019–1022 (1992).

[61] Hu, H. et al. Lipidomics-based insights into the physiological mechanism of wheat in response to heat stress. *Plant Physiol. Biochem.* **205**, 108190 (2023). 37988880 10.1016/j.plaphy.2023.108190

[62] Rahman, M. A. et al. LC-HRMS based non-targeted metabolomic profiling of wheat (Triticum aestivum L.) under post-anthesis drought stress. *Am. J. Plant Sci.* **8**, 3024–3061 (2017).

[63] Selmair, P. L. & Koehler, P. Baking performance of synthetic glycolipids in comparison to commercial surfactants. *J. Agric. Food Chem.* **56**, 6691–6700 (2008). 18636688 10.1021/jf800692b

[64] Qi, Z.-M., Wang, X., Liu, X. & Zhao, J. Asperuloside promotes apoptosis of cervical cancer cells through endoplasmic reticulum stress-mitochondrial pathway. *Chin. J. Integr. Med.* **30**, 34–41 (2024). 37076638 10.1007/s11655-023-3695-z

[65] Kong, Z., Liu, C. & Olatunji, O. J. Asperuloside attenuates cadmium-induced toxicity by inhibiting oxidative stress, inflammation, fibrosis and apoptosis in rats. *Sci. Rep.* **13**, 5698 (2023). 37029128 10.1038/s41598-023-29504-0 PMC10081990

[66] Geisslitz, S., Wieser, H., Scherf, K. A. & Koehler, P. Gluten protein composition and aggregation properties as predictors for bread volume of common wheat, spelt, durum wheat, emmer and einkorn. *J. Cereal Sci.* **83**, 204–212 (2018).

[67] Schall, E. et al. Characterisation and comparison of selected wheat (Triticum aestivum L.) cultivars and their blends to develop a gluten reference material. *Food Chem* **313**, 126049 (2020). 31927320 10.1016/j.foodchem.2019.126049

[68] Wieser, H., Koehler, P. & Scherf, K. A. Chemistry of wheat gluten proteins: Qualitative composition. *Cereal Chem.* **100**, 23–35 (2023).

[69] Morel, M.-H. et al. Insight into gluten structure in a mild chaotropic solvent by asymmetrical flow field-flow fractionation (AsFlFFF) and evidence of non-covalent assemblies between glutenin and ω-gliadin. *Food Hydrocoll.* **103**, 105676 (2020).

[70] Shewry, P. R. & Belton, P. S. What do we really understand about wheat gluten structure and functionality?. *J. Cereal Sci.* **117**, 103895 (2024).

[71] Scherf, K., Brockow, K., Biedermann, T., Koehler, P. & Wieser, H. Wheat-dependent exercise-induced anaphylaxis. *Clin. Exp. Allergy* **46**, 10–20 (2016). 26381478 10.1111/cea.12640

[72] Sollid, L. M. et al. Update 2020: Nomenclature and listing of celiac disease–relevant gluten epitopes recognized by CD4+ T cells. *Immunogenetics* **72**, 85–88 (2020). 31735991 10.1007/s00251-019-01141-w

[73] Norwig, M.-C., Geisslitz, S. & Scherf, K. A. Comparative label-free proteomics study on celiac disease-active epitopes in common wheat, spelt, durum wheat, emmer, and einkorn. *J. Agricult. Food Chem.* **72**(26), 15040–15052 (2024). 10.1021/acs.jafc.4c02657 PMC11228976 38906536

[74] Shewry, P. R. et al. Natural variation in grain composition of wheat and related cereals. *J. Agric. Food Chem.* **61**, 8295–8303 (2013). 23414336 10.1021/jf3054092

