# Supplementary material for: Positive impact of hydroponics and artificial light on yield and quality of wheat
Source: Sci Rep. 2025 Aug 21;15:30768. doi: 10.1038/s41598-025-16204-0 (PMC12370996; doi:10.1038/s41598-025-16204-0)
Supplement: Supplementary file 1 — Supplementary Information. [file 41598_2025_16204_MOESM1_ESM.docx]

**Positive impact of hydroponics and artificial light on yield and quality of wheat**

Simona Bassu^1^, Sebastian Eichelsbacher^1^, Francesco Giunta^2^, Rosella Motzo^2^, Corinna Dawid^3,9^, Martina Gastl^4^, Michael Schloter^5,6^, Katharina A. Scherf^7,8^, Stefan Hör^4^, Yuri Pinheiro Alves De Souza^5^, Stefanie Schulz^5^, Timo D. Stark^9^, Volker Mohler^10^, Senthold Asseng^1*^

^1^Chair of Digital Agriculture, Department of Life Science Engineering, HEF World Agricultural Systems Center, School of Life Sciences, Technical University of Munich, Freising, Germany.

^2^Department of Agricultural Sciences, University of Sassari, Sassari, Italy.

^3^TUM School of Life Sciences, Chemosensory Food Systems, Technical University of Munich, Freising, Germany.

^4^Research Center Weihenstephan for Brewing and Food Quality, Technical University of Munich, Freising, Germany.

^5^Research Unit Comparative Microbiome Analysis, Helmholtz Zentrum München, Neuherberg, Germany.

^6^TUM School of Life Sciences, Professorship of Environmental Microbiology, HEF World Agricultural Systems Center, Technical University of Munich, Freising, Germany.

^7^Leibniz Institute for Food Systems Biology at the Technical University of Munich, Freising, Germany.

^8^TUM School of Life Sciences, Professorship of Food Biopolymer Systems, Technical University of Munich, Freising, Germany.

^9^TUM School of Life Sciences, Food Chemistry and Molecular Sensory Science, Technical University of Munich, Freising, Germany.

^10^Bavarian State Research Center for Agriculture, Freising, Germany.

*Corresponding author: senthold.asseng@tum.de

**Supplementary material**

**Table S1.** **P-values** of key parameters obtained from Welch’s t-test comparing pairs of treatments. A significance threshold of α = 0.05 was used to determine statistical significance.

| **p-value** | **Yield** | **Grain number** | **Grain weight** |
| --- | --- | --- | --- |
| Other cultivars-Apogee | 0.1499 | 0.0334 | 4.72E-03 |
| Other cultivars-L | 5.64E-05 | 2.00E-09 | 6.81E-05 |
| Other cultivars-M | 2.57E-07 | 0.002744 | 0.003857 |
| Other cultivars-H | 2.34E-06 | 1.47E-06 | 8.81E-07 |
| Apogee-L | 6.84E-07 | 3.02E-08 | 0.09204 |
| Apogee-M | 0.0003091 | 0.003465 | 0.02852 |
| Apogee-H | 3.52E-06 | 2.67E-06 | 0.007342 |
| L-M | 9.58E-11 | 0.006505 | 0.09613 |
| L-H | 6.14E-06 | 3.51E-06 | 0.008353 |
| M-H | 0.005194 | 0.2342 | 0.3849 |


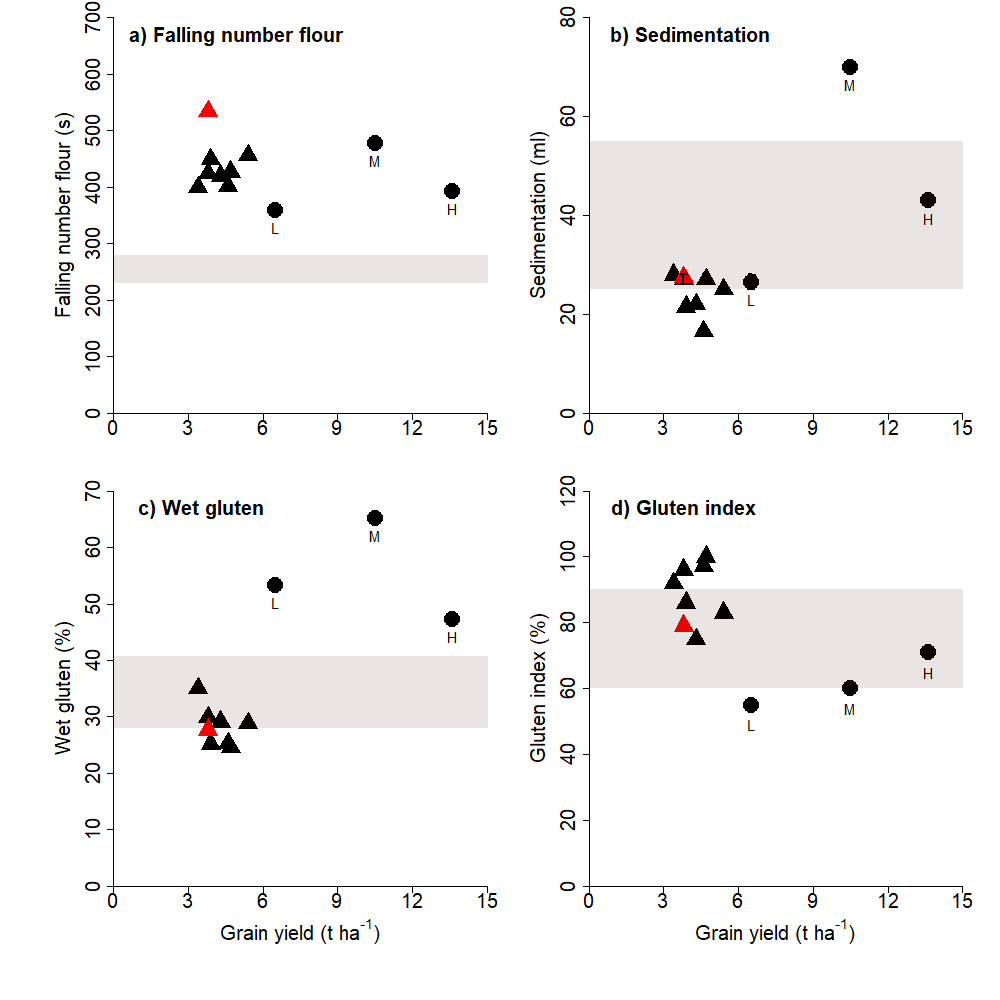


**Figure S1. Grain yield quality.** (a) Falling number (s), (b) sedimentation (ml), (c) wet gluten concentration (%) and (d) gluten index (%) in the grain versus grain yield for field measurements (triangles) and indoor measurements (full circles). The red symbol indicates the cultivar Apogee grown in the field. For (a) and (b) the grey shaded area indicates the minimum range needed for bread-making quality^1-5^. For (c) and (d) the grey area indicates the observed range from literature ^6^, as there is no minimum range defined. Note, data are based on two technical replications from a mixed sample of each treatment. Vertical bars are standard errors, visible when exceeding the size of the symbol. There are no replicates for falling number, for sedimentation for indoor M – medium yielding and H – high yielding experiment, wet gluten and gluten index.


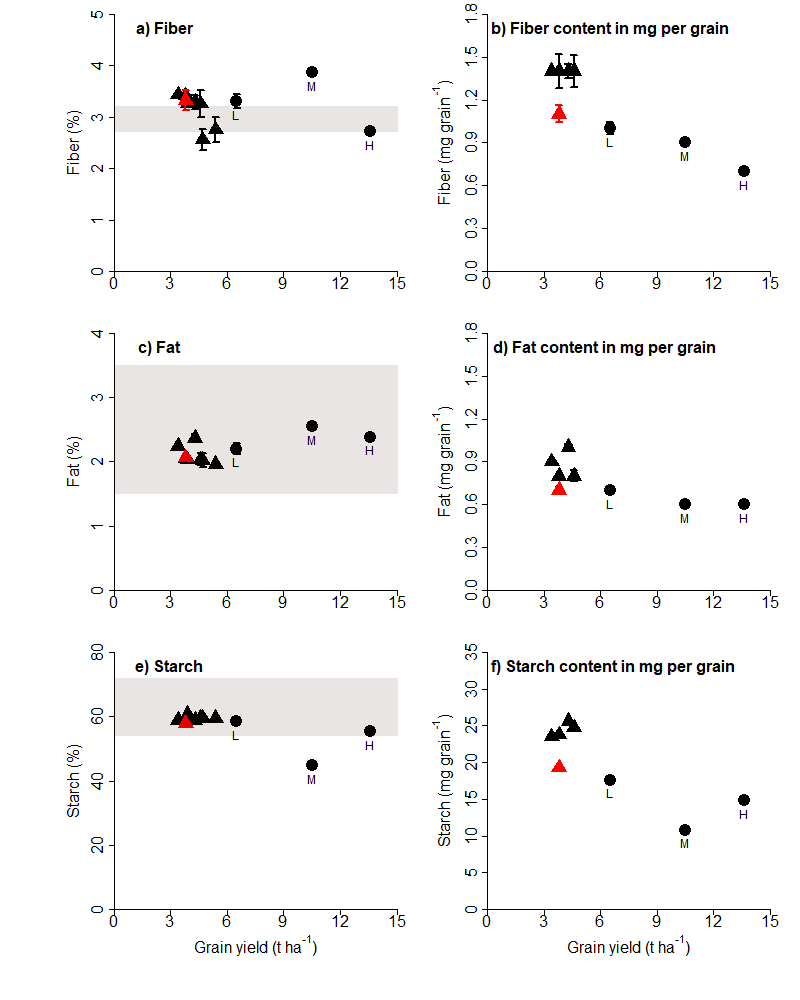


**Figure S2. Grain yield quality.** (a, c, e) Concentration (%) and (b, d, f) weight per average grain (mg grain^-1^) for (a, b) fiber, (c, d) fat, and (e, f) starch for field measurements (triangles) and indoor measurements (full circles). The red symbol indicates the cultivar Apogee grown in the field. The grey area indicates the observed range from literature^7-9^. Note, data are based on two technical replications from a mixed sample of each treatment. Vertical bars are standard errors, visible when exceeding the size of the symbol.


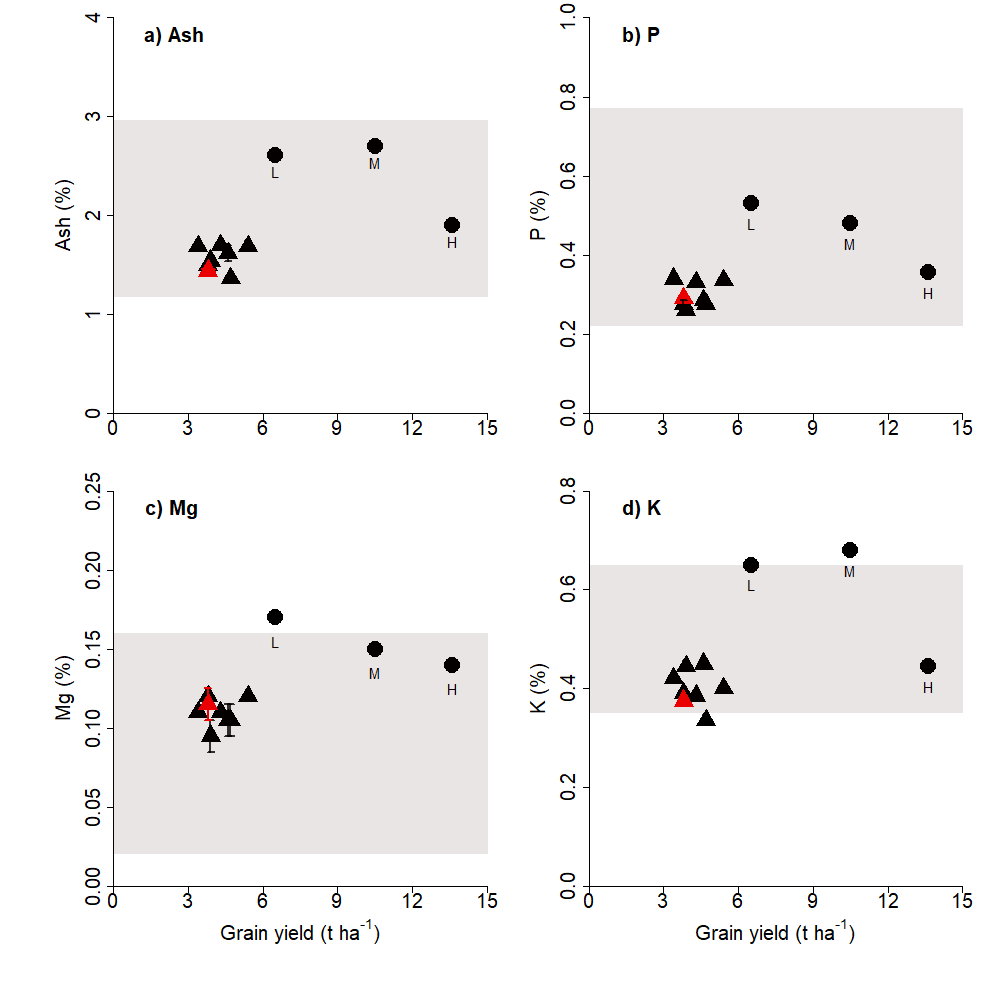


**Figure S3. Grain yield minerals.** (a) Ash (%), (b) phosphorus (%), (c) magnesium (%), and (d) potassium (%) in the grain versus grain yield for field measurements (triangles) and indoor measurements (full circles). The red symbol indicates the cultivar Apogee grown in the field. The grey shaded area indicates the observed range from literature^10^. Note, data are based on two technical replications from a mixed sample of each treatment. Vertical bars are standard errors, visible when exceeding the size of the symbol.


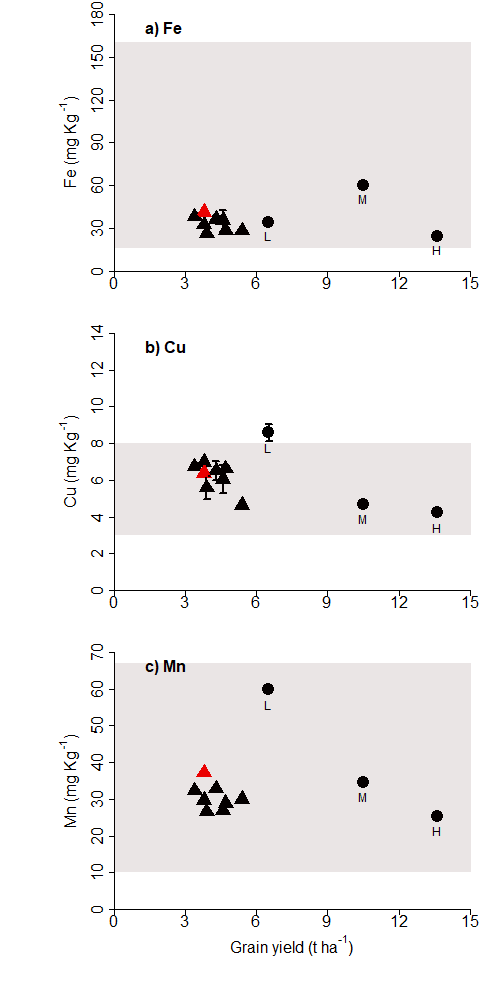


**Figure S4. Grain yield minerals**. (a) Iron (mg kg^-1^), (b) copper (mg kg^-1^), and (c) manganese (mg kg^-1^) in the grain versus grain yield for field measurements (triangles) and indoor measurements (full circles). The red symbol indicates the cultivar Apogee grown in the field. The grey shaded area indicates the observed range from literature^10^. Note, data are based on two technical replications from a mixed sample of each treatment. Vertical bars are standard errors, visible when exceeding the size of the symbol.

**
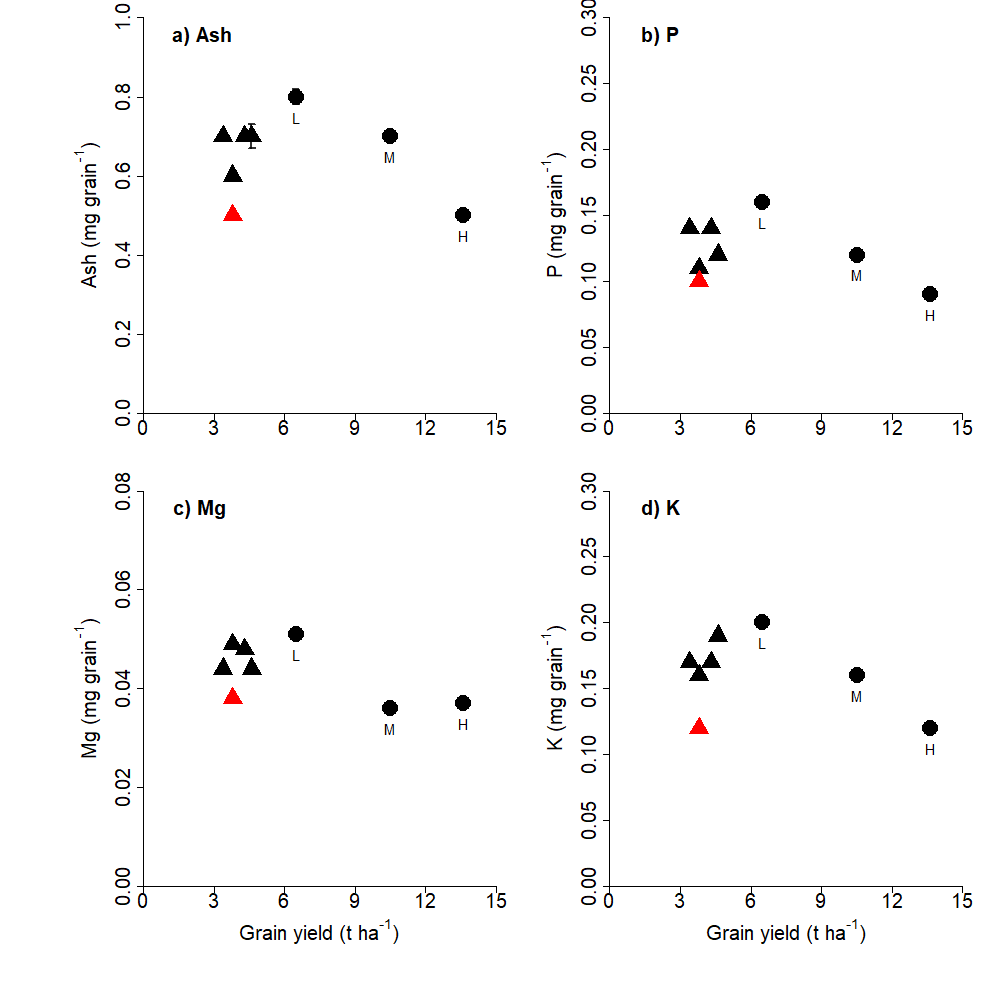
**

**Figure S5. Mineral content per grain** (mg grain^-1^) for (a) ash, (b) phosphorus, (c) magnesium, and (d) potassium versus grain yield for field measurements (triangles) and indoor measurements (full circles). The red symbol indicates the cultivar Apogee grown in the field. Note, data are based on two technical replications from a mixed sample of each treatment. Vertical bars are standard errors, visible when exceeding the size of the symbol.

**
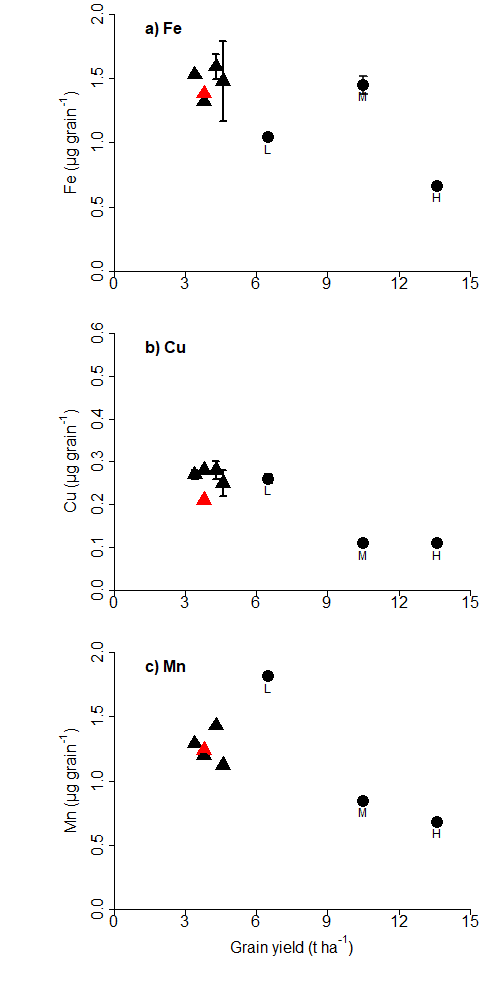
**

**Figure S6.** **Mineral content per grain** (μg grain^-1^) for (a) iron, (b) copper, and (c) manganese versus grain yield for field measurements (triangles) and indoor measurements (full circles). The red symbol indicates the cultivar Apogee grown in the field. Note, data are based on two technical replications from a mixed sample of each treatment. Vertical bars are standard errors, visible when exceeding the size of the symbol.

**
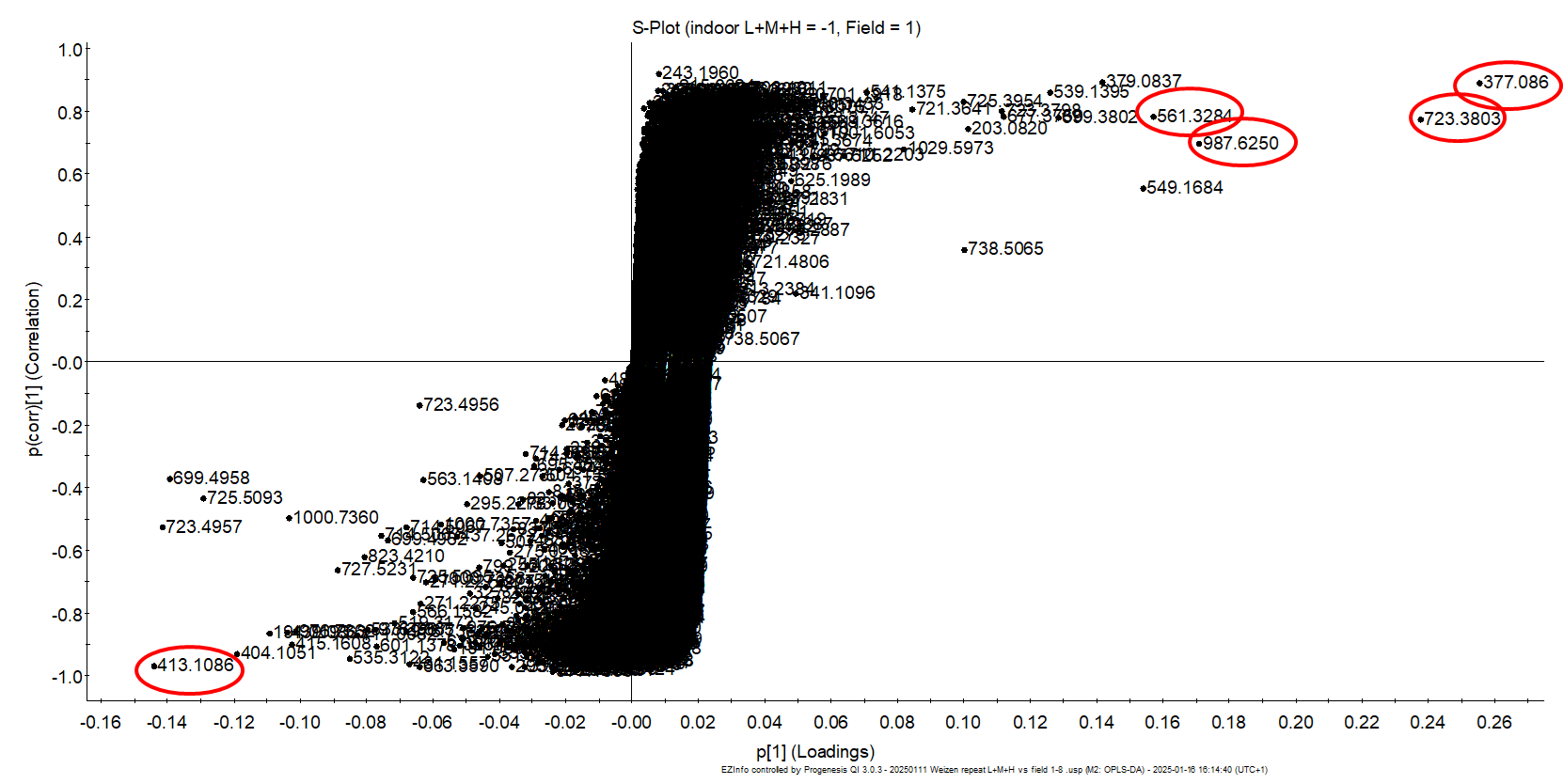
**

**Figure S7.** S-plot of wheat grain comparing metabolites from L, M, H indoor experiments versus cultivars grown in the field (F). m/z with highest contribution as well as significance on the difference of both groups are highlighted in red.

**
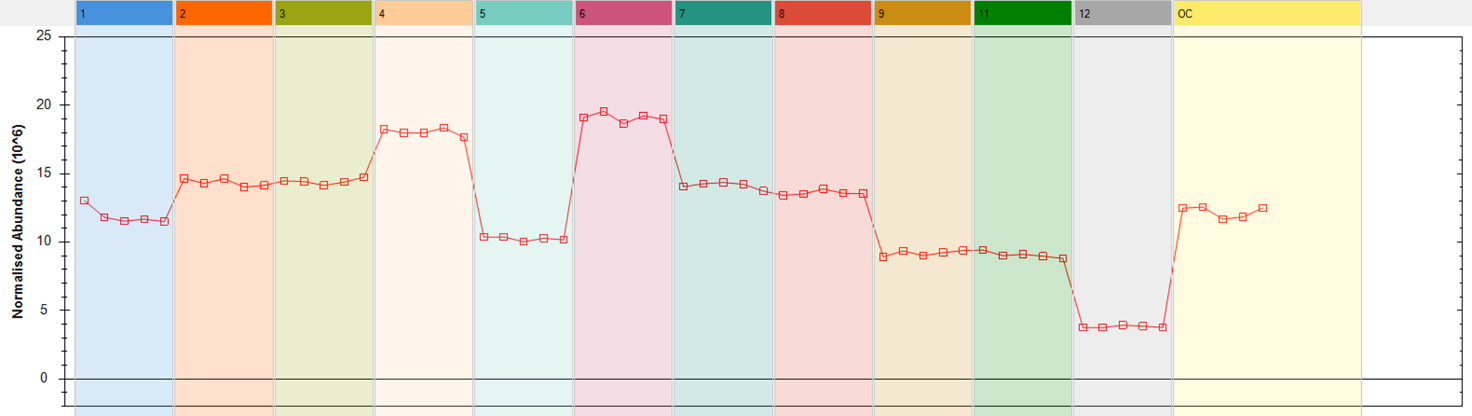
**

**Figure S8.** Trend plot of the compound as formic acid adduct with retention time 6.42_724.3876n (m/z 723.3818): 1-linoleoyl-3-*O*-(*β*-D-digalactopyranosyl)*-*glycerol (DGMG-L), for cultivars in the field (1-8), including cultivar Apogee in the field (5), and cultivar Apogee grown indoors, for L – low-yielding (9), M – medium yielding (12) and H – high yielding experiment (11).


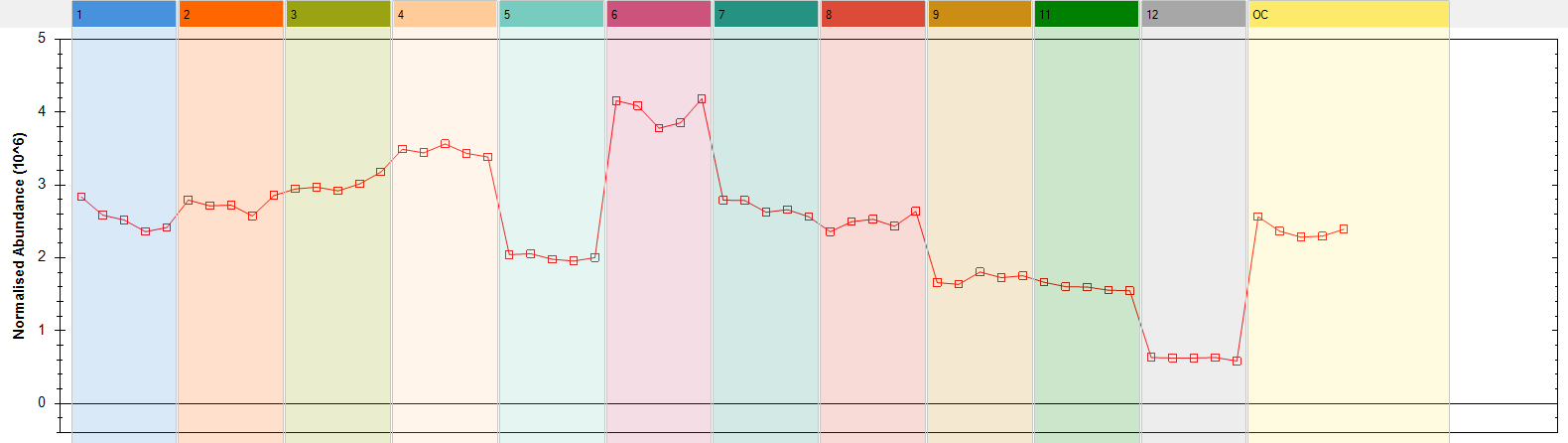


**Figure S9.** Trend plot of the compound with retention time 6.42_m/z 677.3750: 1-linoleoyl-3-*O*-(*β*-D-digalactopyranosyl)*-*glycerol (DGMG-L), for cultivars in the field (1-8), including cultivar Apogee in the field (5), and cultivar Apogee grown indoors, for L – low yielding (9), M – medium yielding (12) and H – high yielding experiment (11).

**
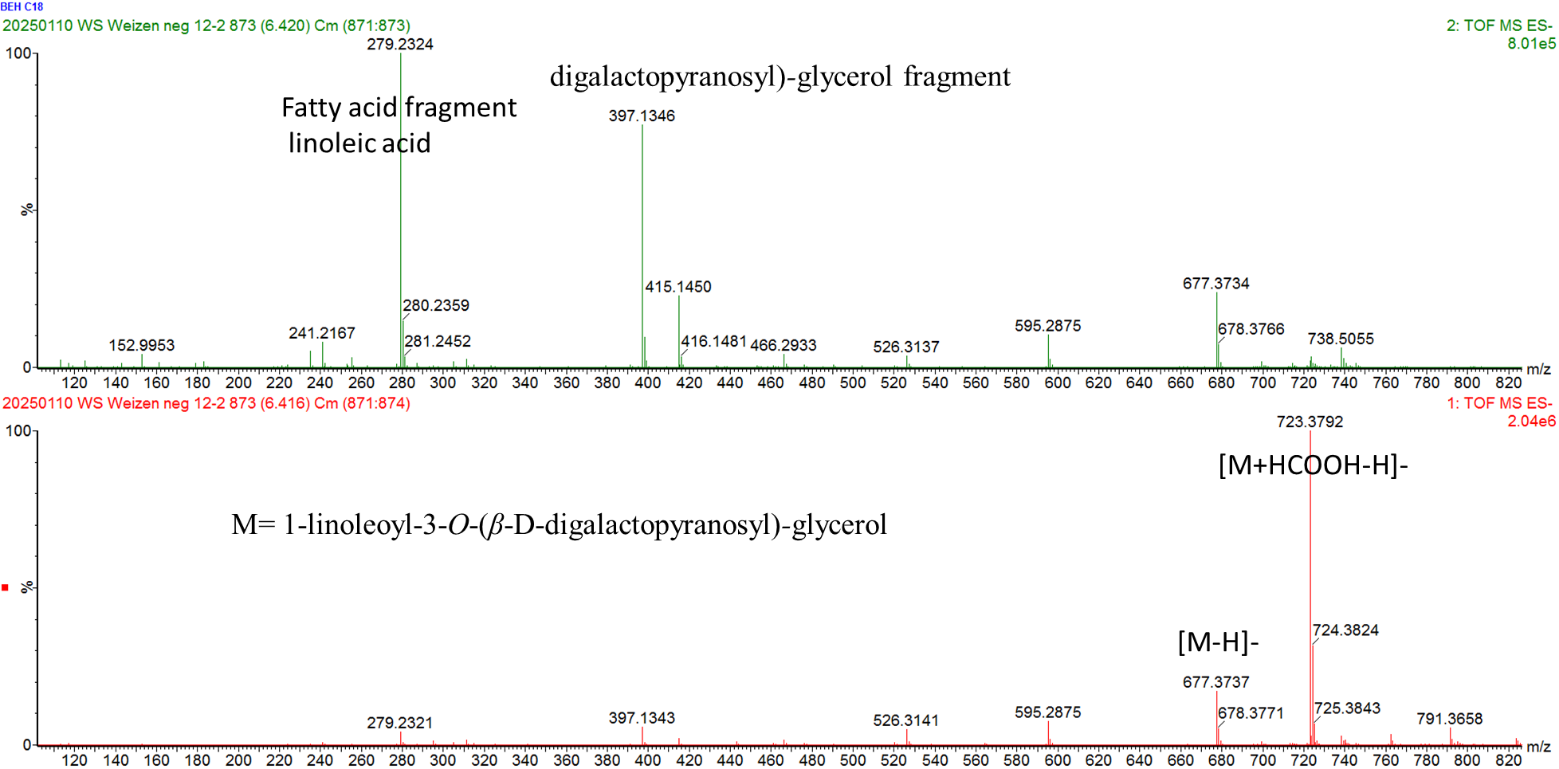
**

**Figure S10.** High resolution electrospray Ionisation mass spectrometry **(**HRESIMS) (lower panel, low collision energy) and MS^e^ (upper panel, high collision energy) spectrum (neg.) of the compound with the retention time 6.42_724.3876n (m/z 723.3818 as formic acid adduct and m/z 677.3750 as M-H-): 1-linoleoyl-3-*O*-(*β*-D-digalactopyranosyl)*-*glycerol (DGMG-L).

**
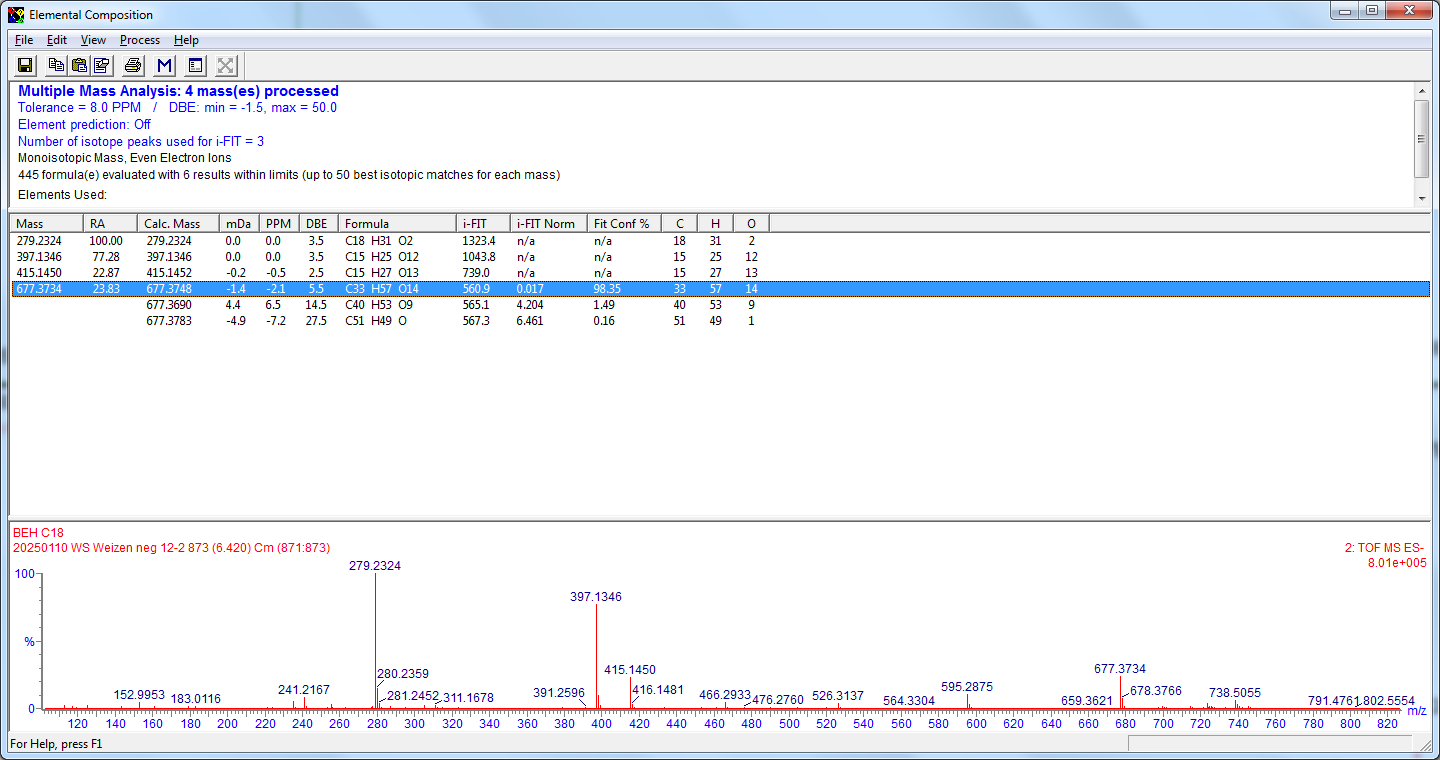
**

**Figure S11.** Elemental Composition Report for the compound with the retention time 6.42_724.3876n (m/z 723.3818 as formic acid adduct and m/z 677.3750 as M-H-): 1-linoleoyl-3-*O*-(*β*-D-digalactopyranosyl)*-*glycerol (DGMG-L).


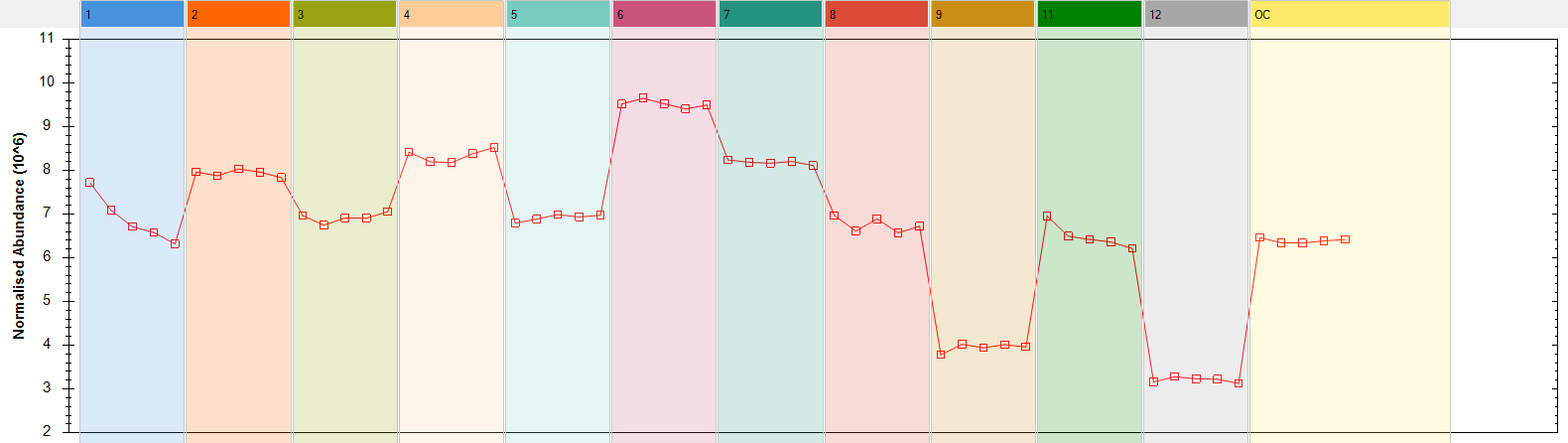


**Figure S12.** Trend plot of the compound with retention time 7.21_516.3302n (m/z 515.3235): 1-linoleoyl-3-*O*-(*β*-D-galactopyranosyl)*-*glycerol (MGMG-L), for cultivars in the field (1-8), including cultivar Apogee in the field (5), and cultivar Apogee grown indoors, for L – low yielding (9), M – medium yielding (12) and H – high yielding experiment (11).


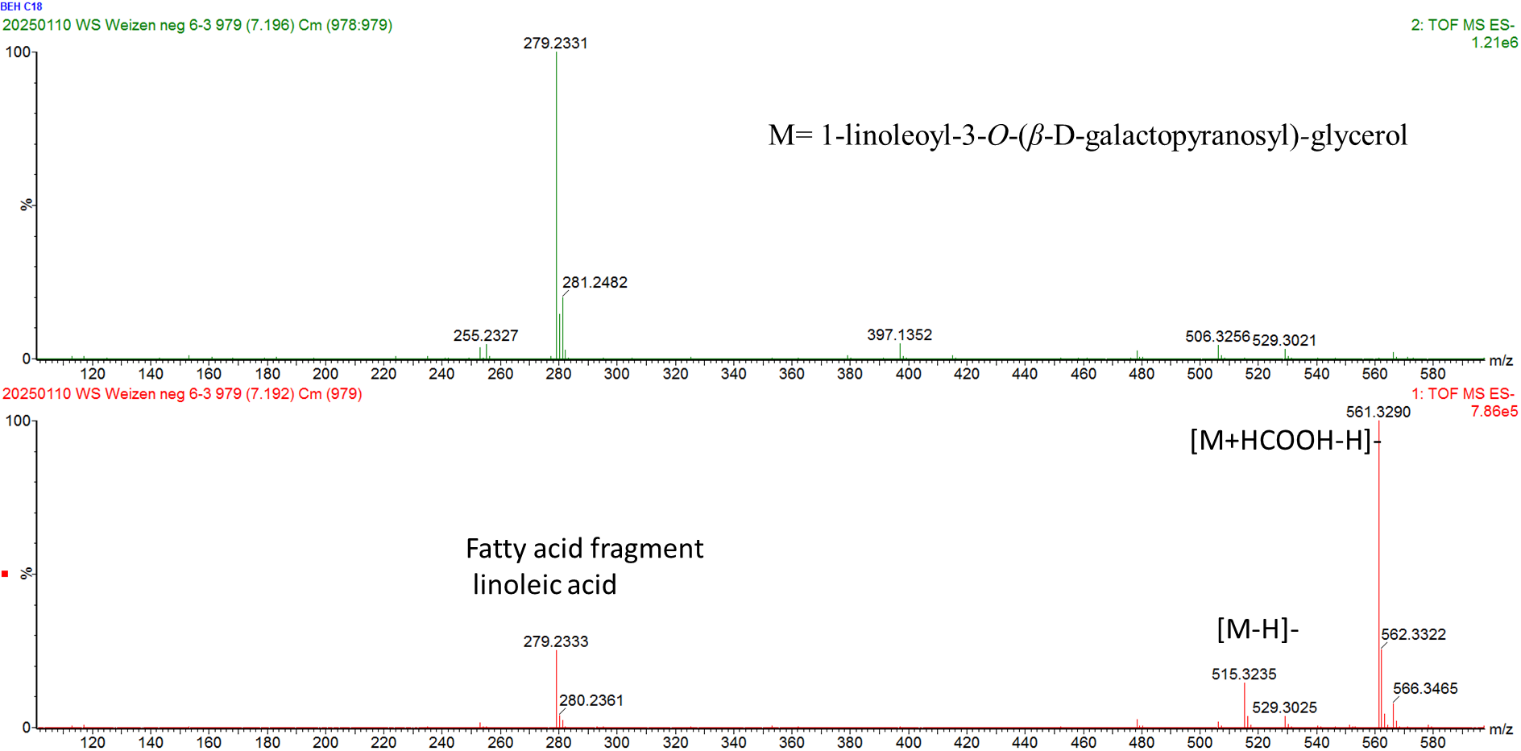


**Figure S13.** High resolution electrospray Ionisation mass spectrometry **(**HRESIMS) (lower panel, low collision energy) and MS^e^ (upper panel, high collision energy) spectrum (neg.) of the compound with the retention time 7.21_516.3302n (m/z 515.3235) and m/z 561.3284 as formic acid adduct: 1-linoleoyl-3-*O*-(*β*-D-galactopyranosyl)*-*glycerol (MGMG-L).


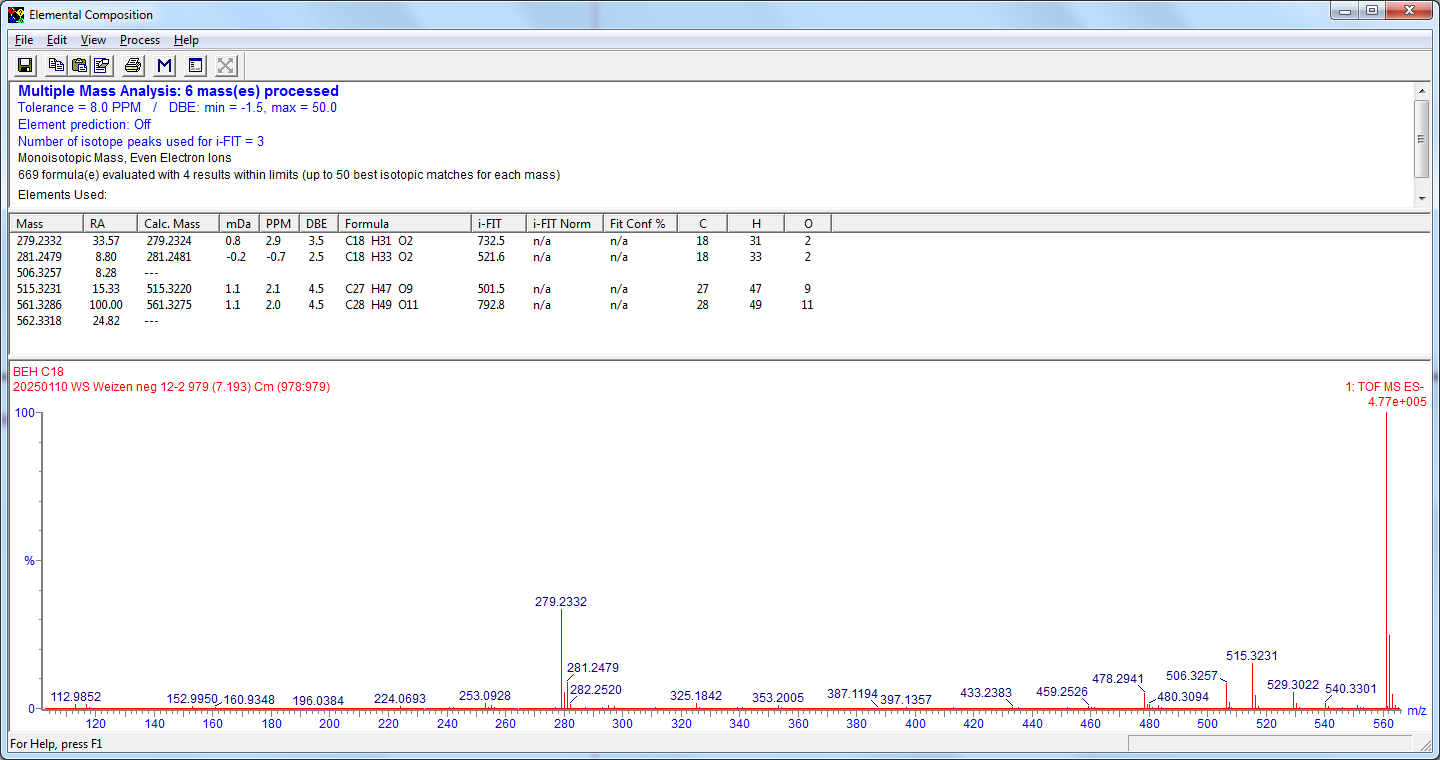


**Figure S14.** Elemental Composition Report for the compound with the retention time 7.21_516.3302n (m/z 515.3235) and m/z 561.3284 as formic acid adduct: 1-linoleoyl-3-*O*-(*β*-D-galactopyranosyl)*-*glycerol (MGMG-L).


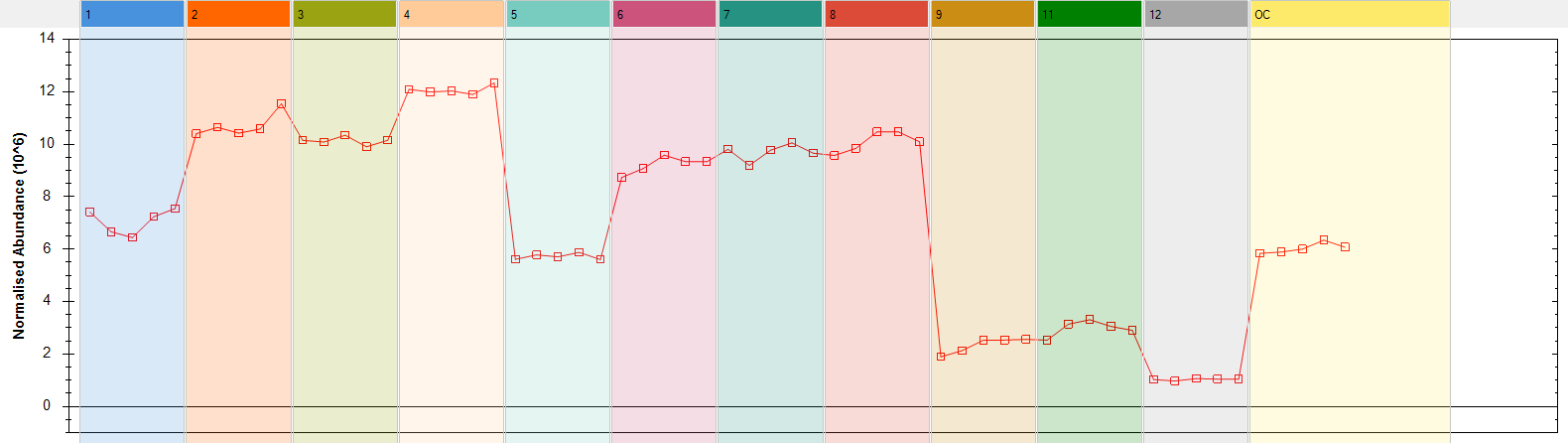


**Figure S15.** Trend plot of the compound with retention time 0.97_378.0934n (m/z 377.3235): trehalose (maltose, sucrose), for cultivars in the field (1-8), including cultivar Apogee in the field (5), and cultivar Apogee grown indoors, for L – low yielding (9), M – medium yielding (12) and H – high yielding experiment (11).

**
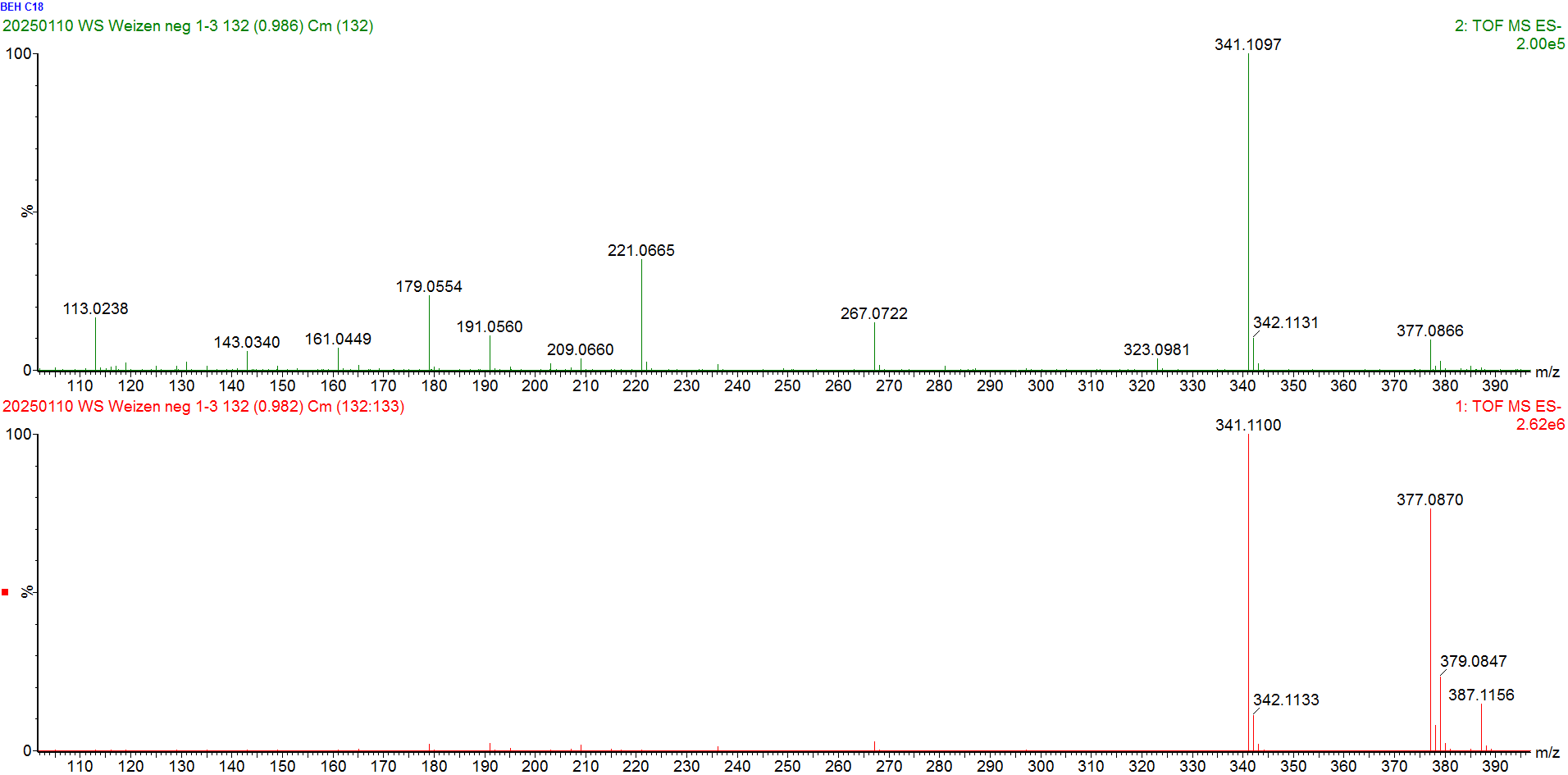
**

**Figure S16.** High resolution electrospray Ionisation mass spectrometry **(**HRESIMS) (lower panel, low collision energy) and MS^e^ (upper panel, high collision energy) spectrum (neg.) of the compound with the retention time 0.97_378.0934n (m/z 377.3235): trehalose (maltose, sucrose).


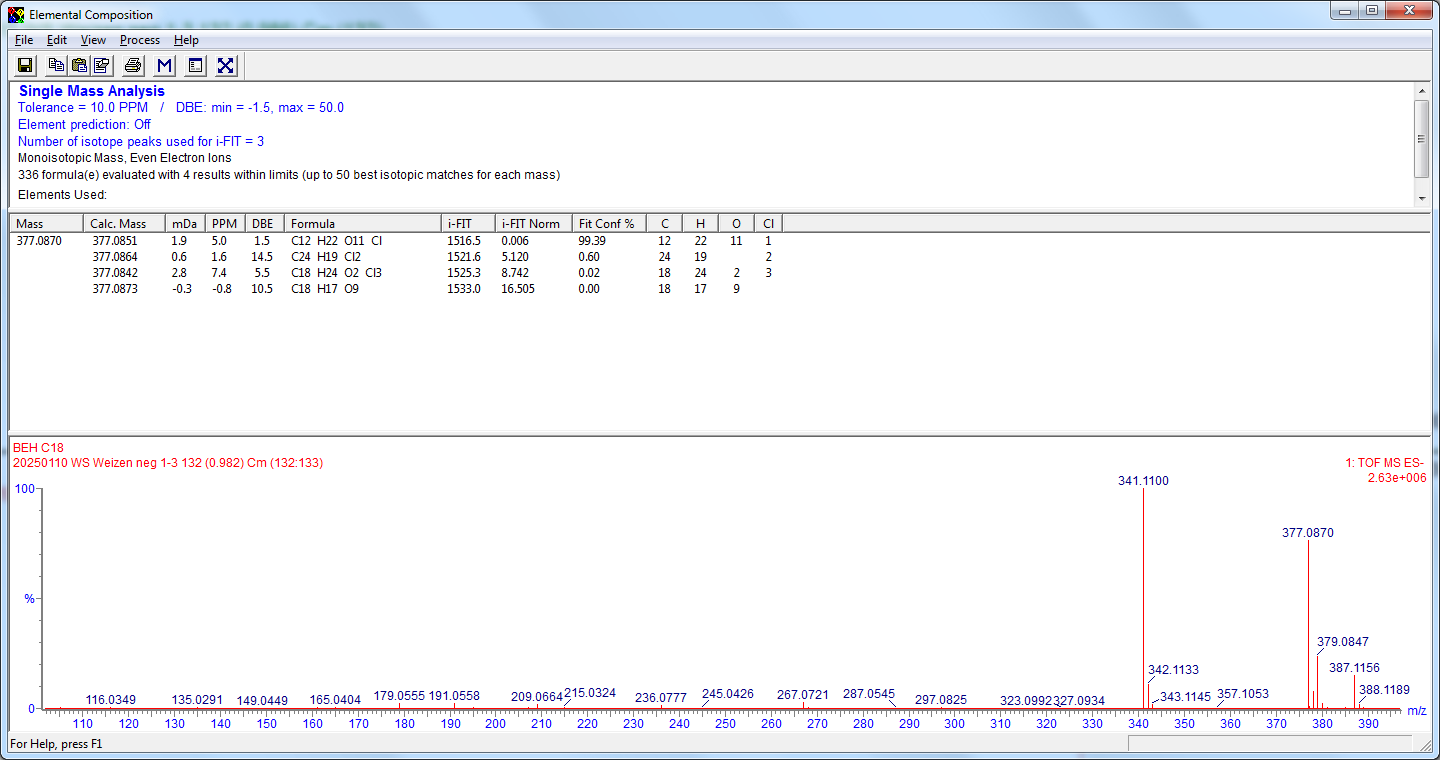


**
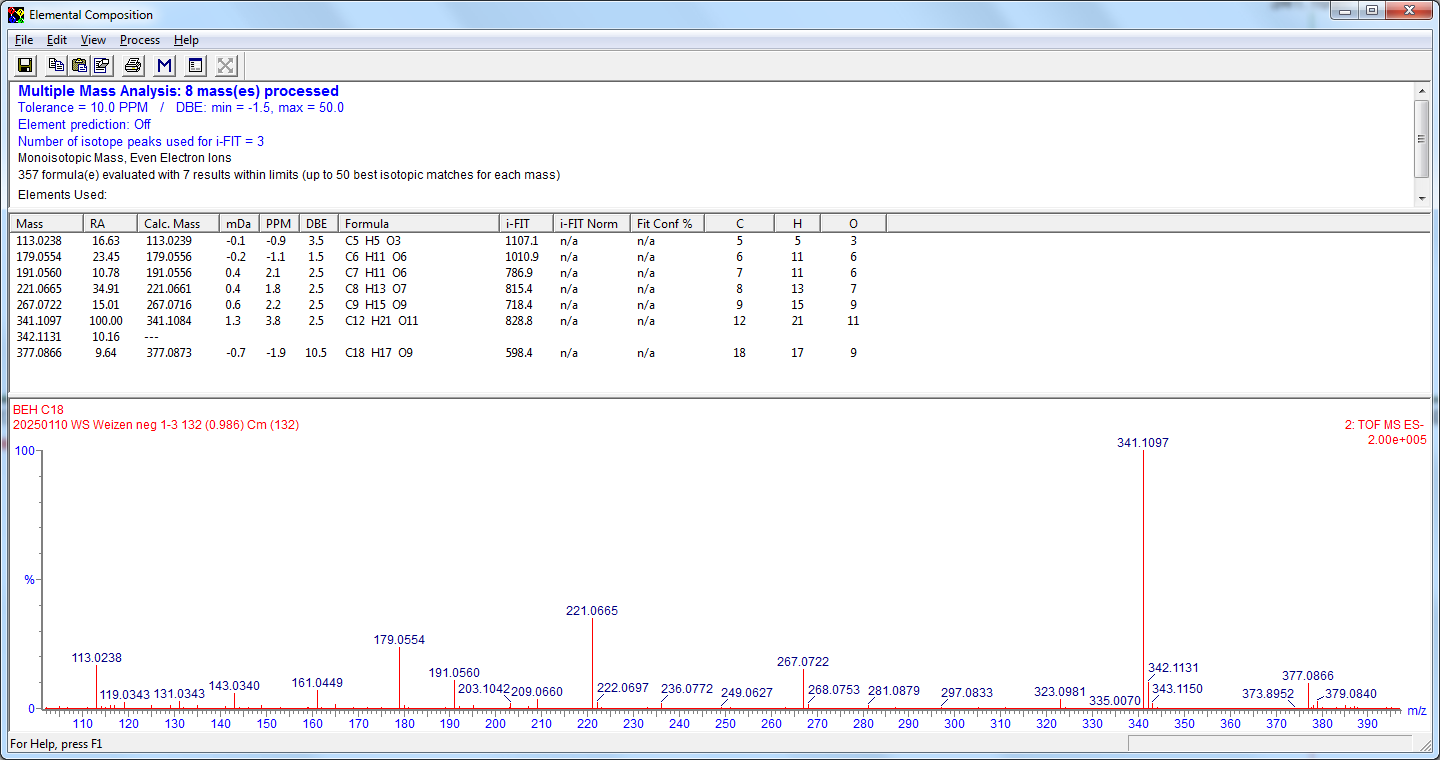
**

**Figure S17.** Elemental composition report for the compound with the retention time 0.97_378.0934n (m/z 377.3235): trehalose (maltose, sucrose).


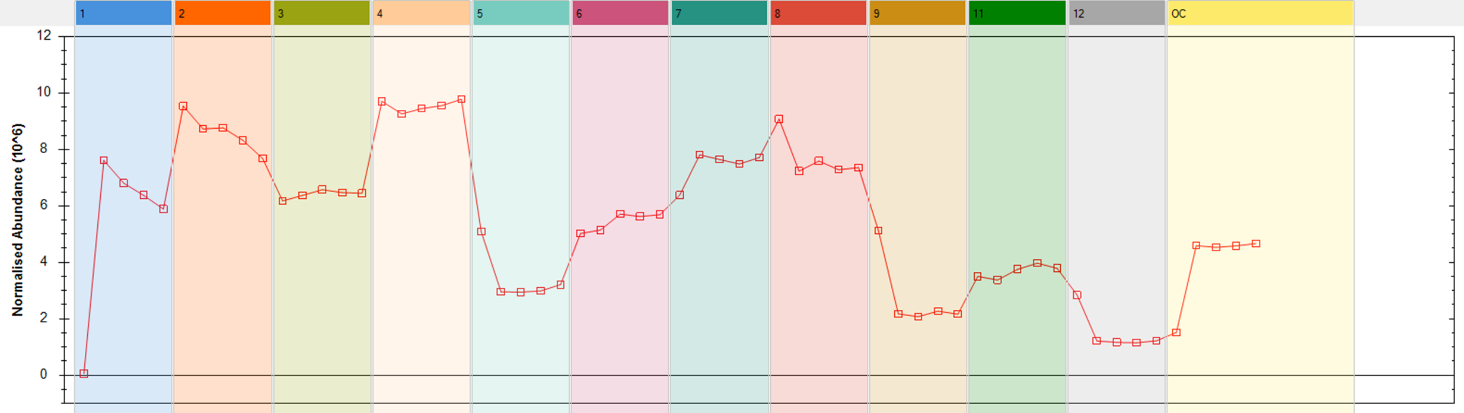


**Figure S18.** Trend plot of the compound with retention time 7.51_942.6268n (m/z 987.6250 as formic acid adduct): 1-oleoyl-2-linoleoyl-3-*O*-(*β*-D-digalactopyranosyl)-*sn-*glycerol (DGDG-OL) or 1-linoleoyl-2-oleoyl-3-*O*-(*β*-D-digalactopyranosyl)-*sn-*glycerol (DGDG-LO), for cultivars in the field (1-8), including cultivar Apogee in the field (5), and cultivar Apogee grown indoors, for L – low yielding (9), M – medium yielding (12) and H – high yielding experiment (11).


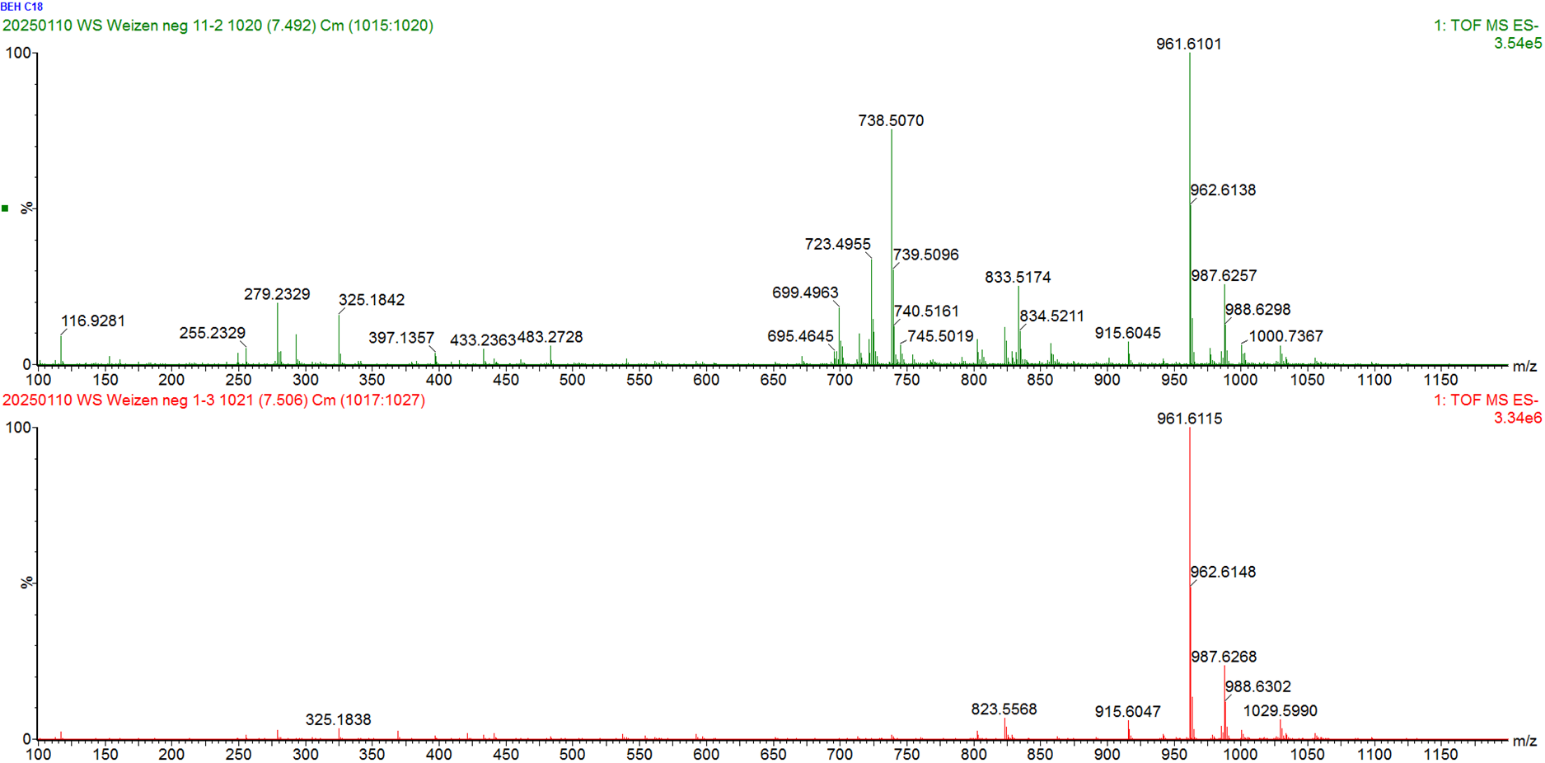


**Figure S19.** High resolution electrospray Ionisation mass spectrometry **(**HRESIMS) (lower panel, low collision energy) and MS^e^ (upper panel, high collision energy) spectrum (neg.) of the compound with the retention time 7.51_942.6268n (m/z 987.6250 as formic acid adduct): 1-oleoyl-2-linoleoyl-3-*O*-(*β*-D-digalactopyranosyl)-*sn-*glycerol (DGDG-OL) or 1-linoleoyl-2-oleoyl-3-*O*-(*β*-D-digalactopyranosyl)-*sn-*glycerol (DGDG-LO).


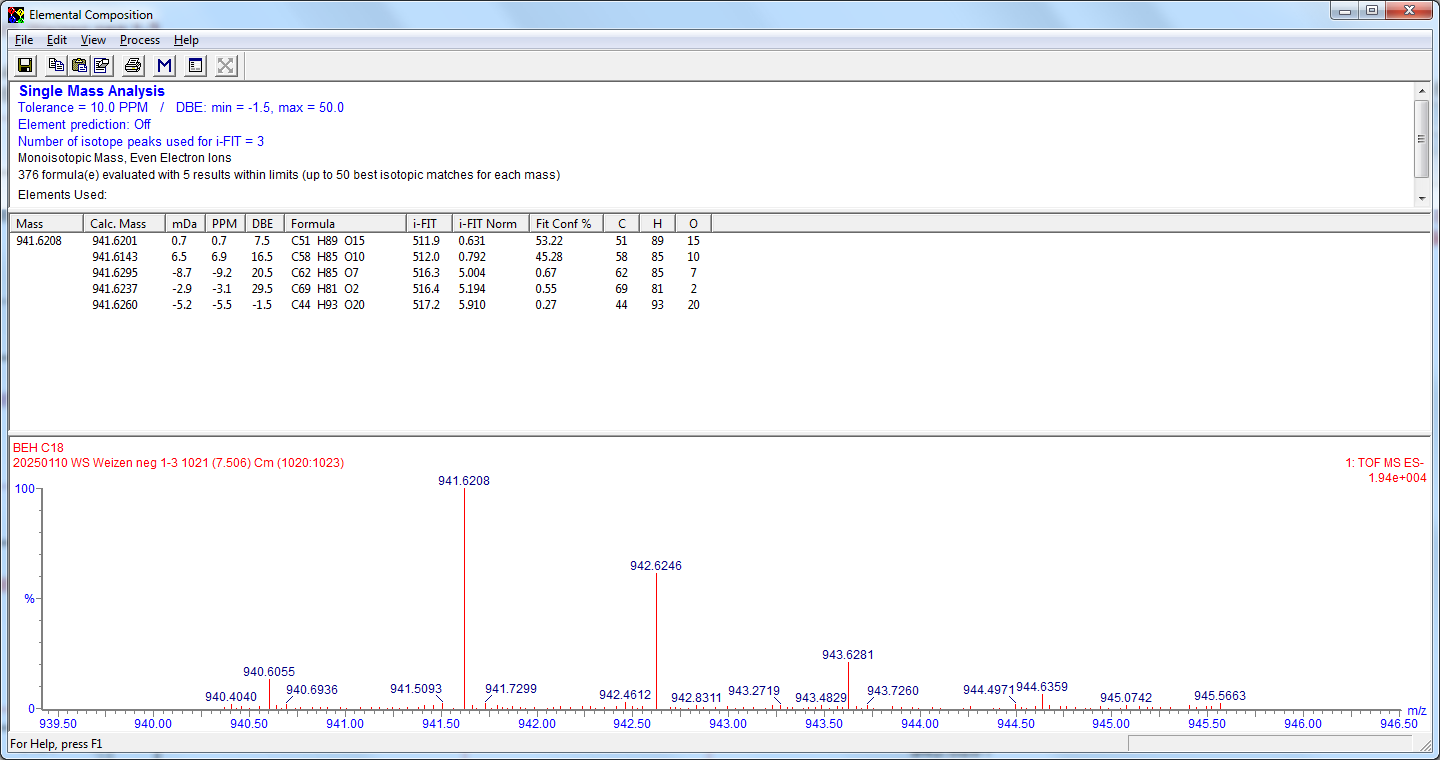


**Figure S20.** Elemental composition report for the compound with the retention time 7.51_942.6268n (m/z 987.6250 as formic acid adduct): 1-oleoyl-2-linoleoyl-3-*O*-(*β*-D-digalactopyranosyl)-*sn-*glycerol (DGDG-OL) or 1-linoleoyl-2-oleoyl-3-*O*-(*β*-D-digalactopyranosyl)-*sn-*glycerol (DGDG-LO).

**
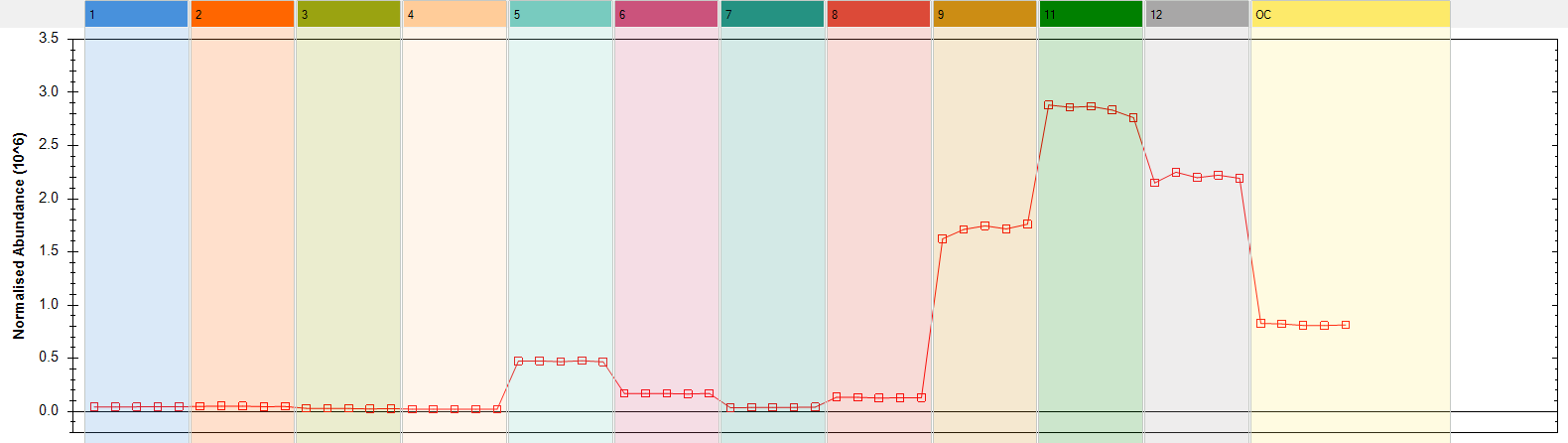
**

**Figure S21.** Trend plot of the compound with retention time 2.76 min_414.1159n (m/z 413.1086): asperuloside), for cultivars in the field (1-8), including cultivar Apogee in the field (5), and cultivar Apogee grown indoor, for L – low yielding (9), M – medium yielding (12) and H – high yielding (11).

**
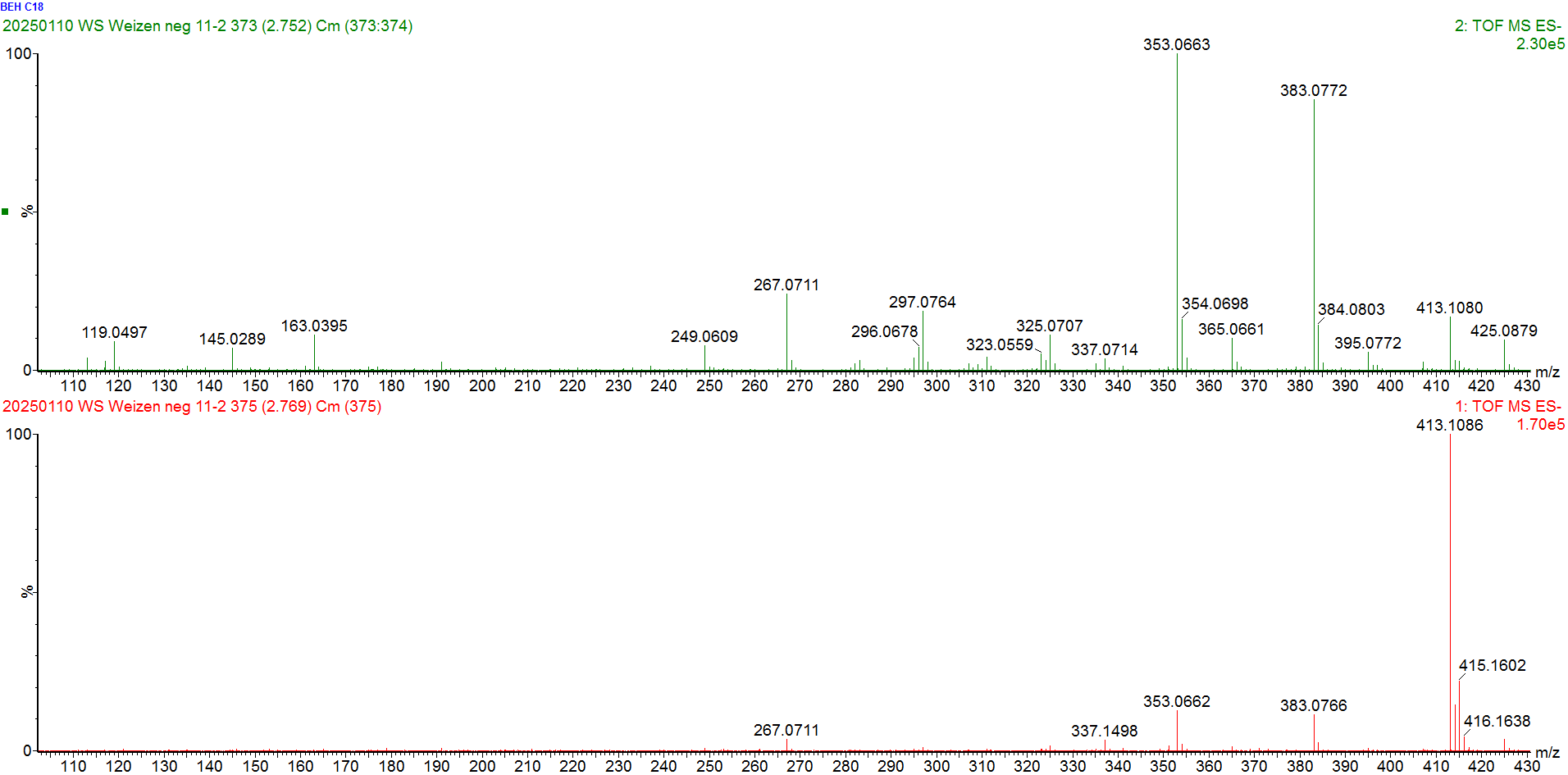
**

**Figure S22.** High resolution electrospray Ionisation mass spectrometry **(**HRESIMS) (lower panel, low collision energy) and MS^e^ (upper panel, high collision energy) spectrum (neg.) of the compound with the retention time 2.76 min_414.1159n (m/z 413.1086): asperuloside.


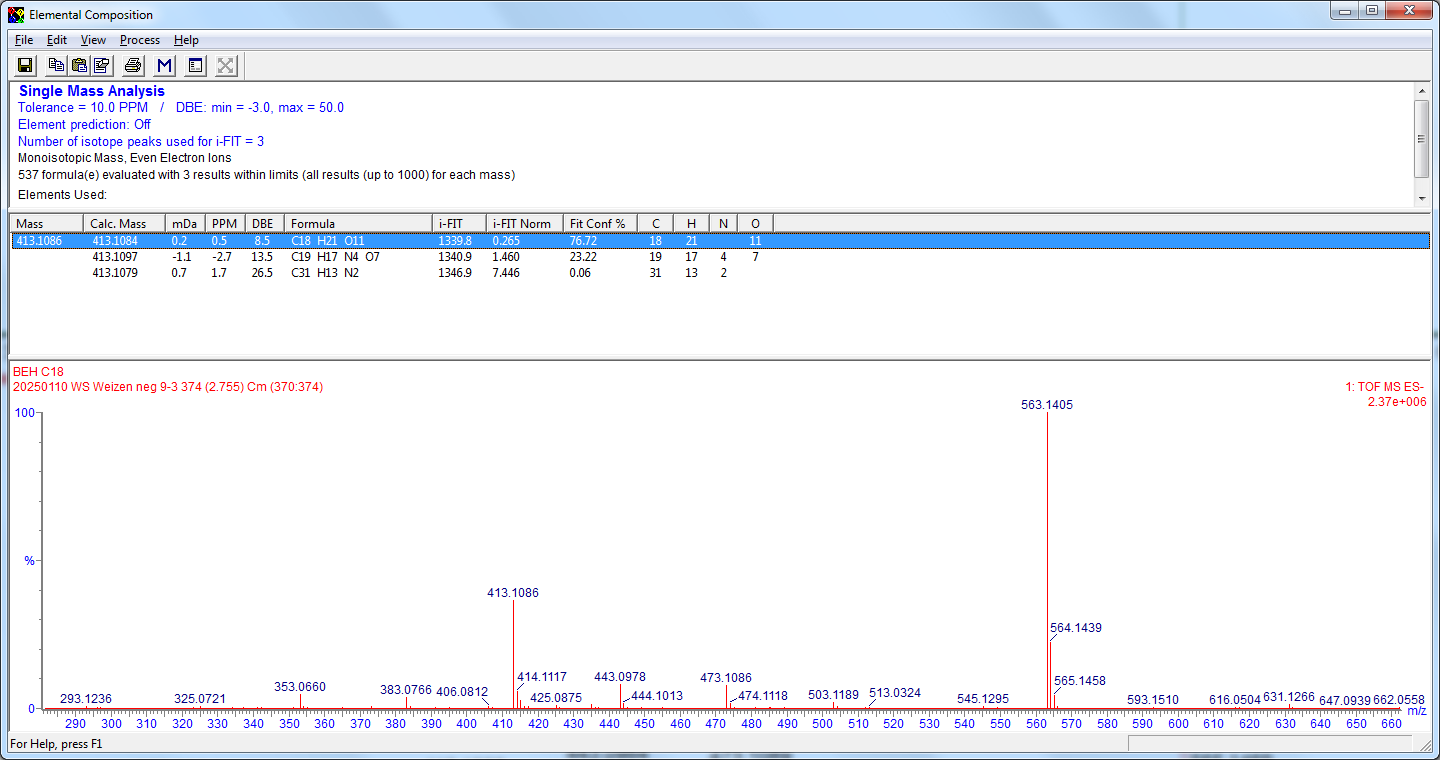


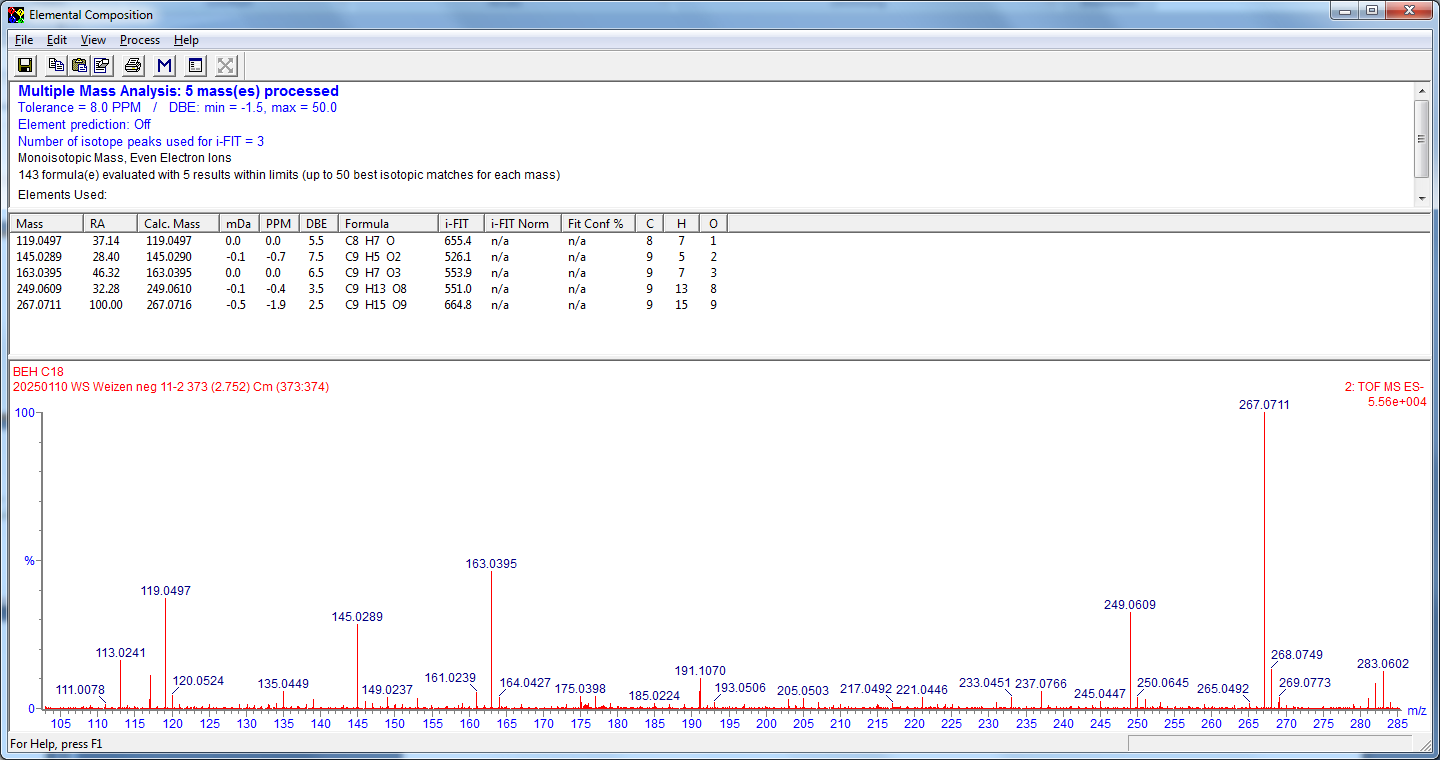


**Figure S23.** Elemental composition report for the compound with the retention time 2.76 min_414.1159n (m/z 413.1086): asperuloside.


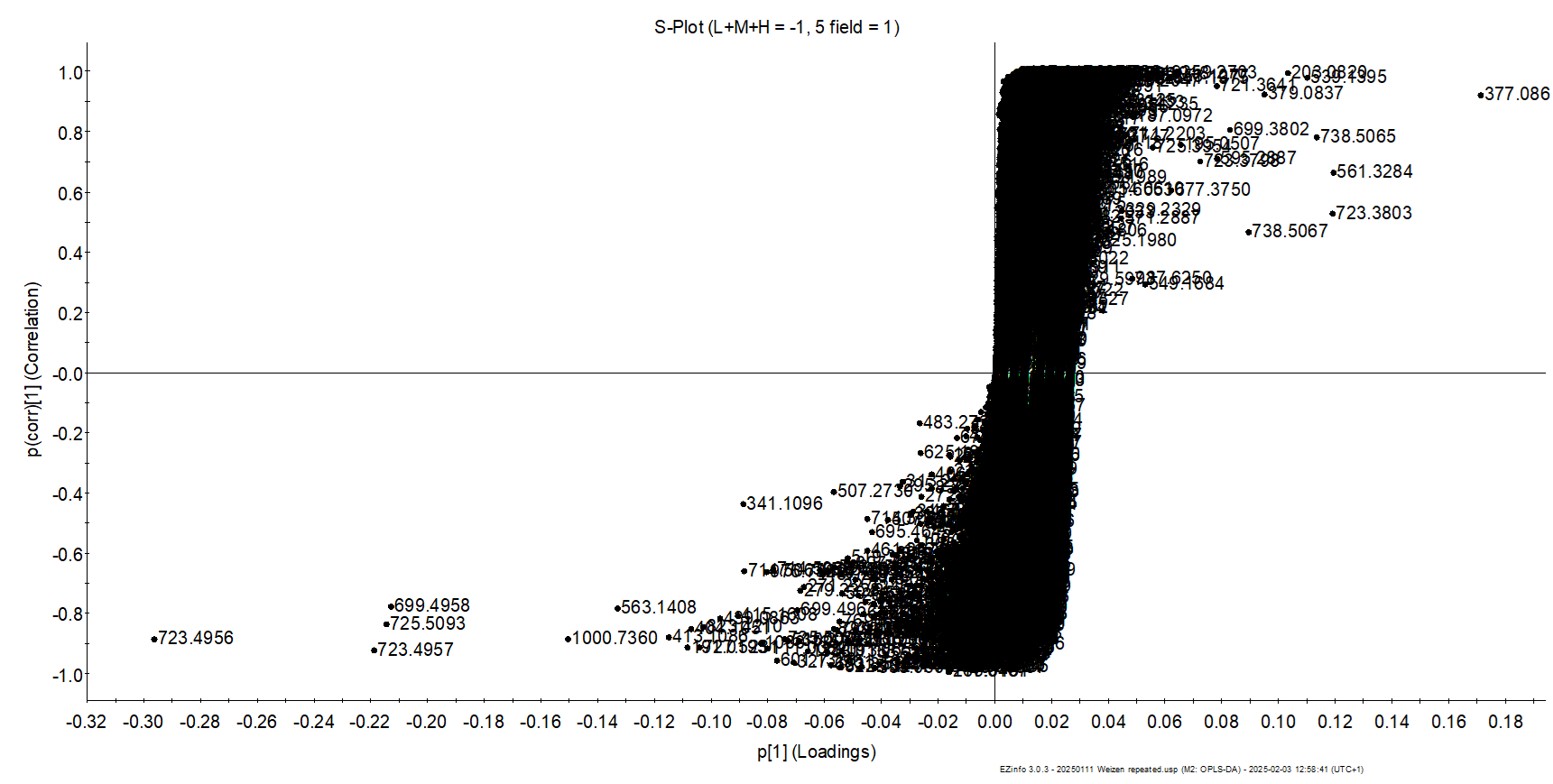


**Figure S24.** S-plot of wheat grain comparing metabolites from L+M+H indoor experiments versus cultivar apogee (red) grown in the field (F).


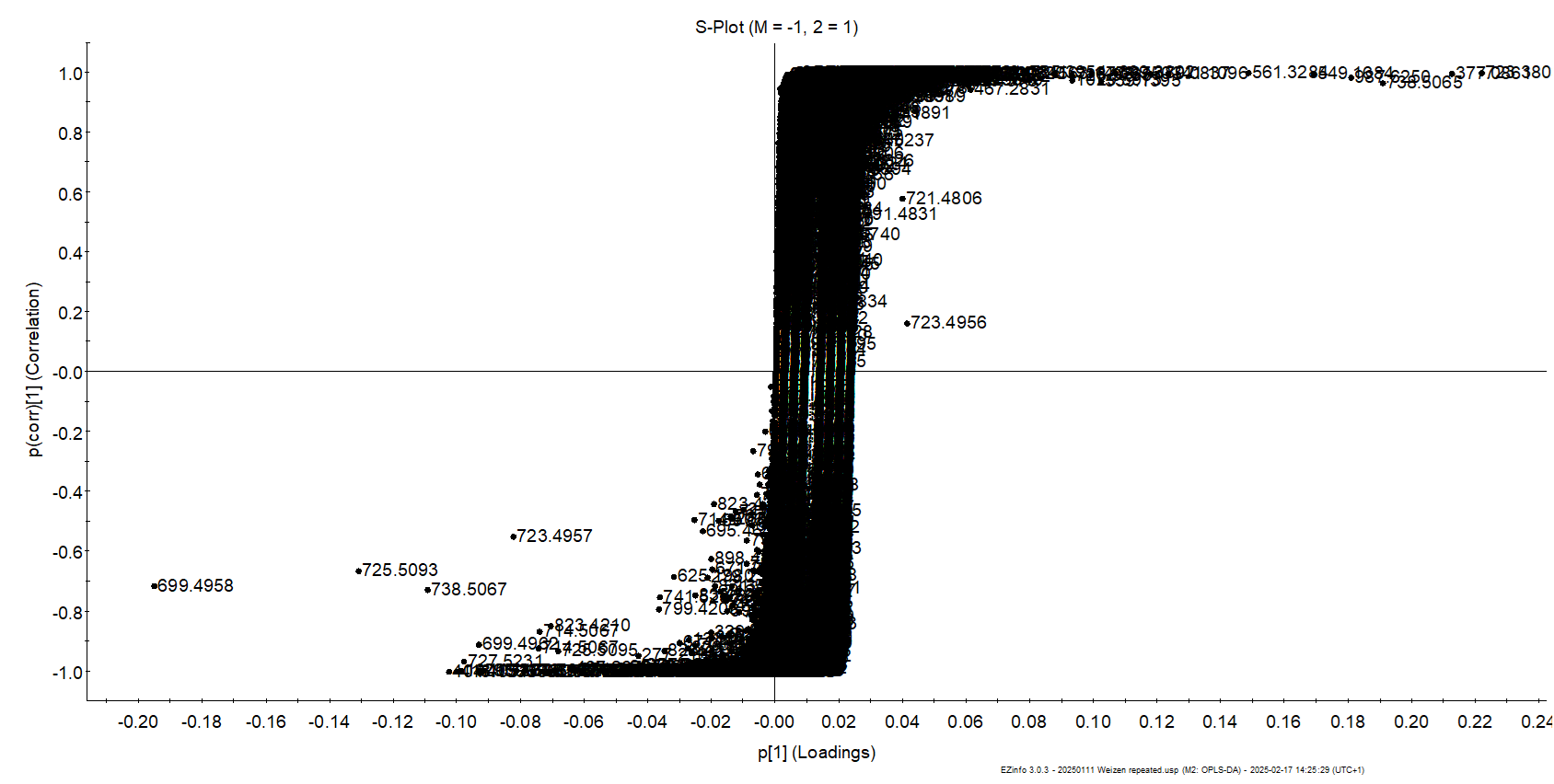


**Figure S25.** S-plot of wheat grain comparing metabolites from M indoor experiments versus cultivar Torril (2) in the field; both groups showing the highest separation on PC1.

**
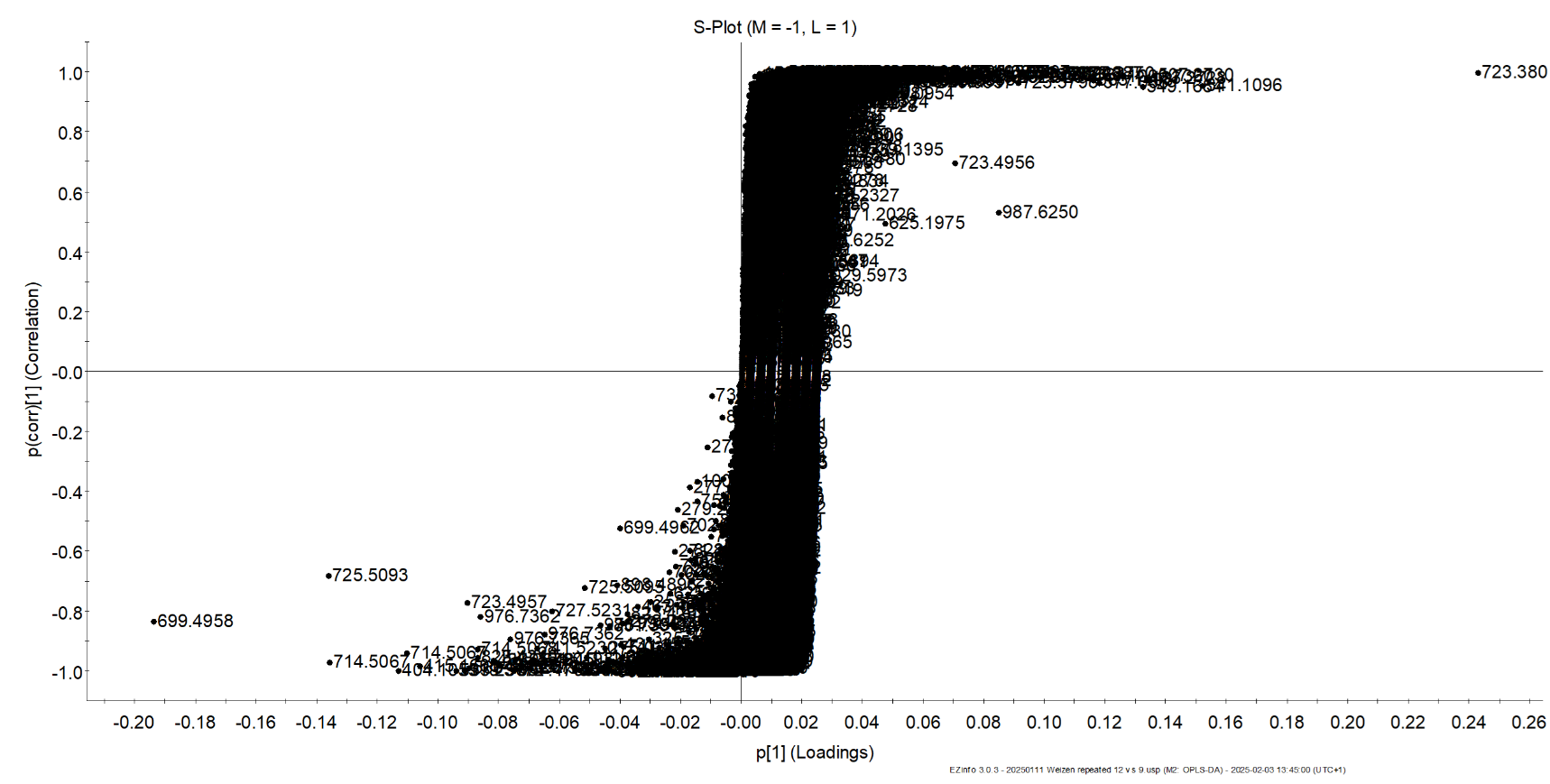
**

**Figure S26.** S-plot of wheat grain comparing metabolites from apogee M versus L grown indoors.

**
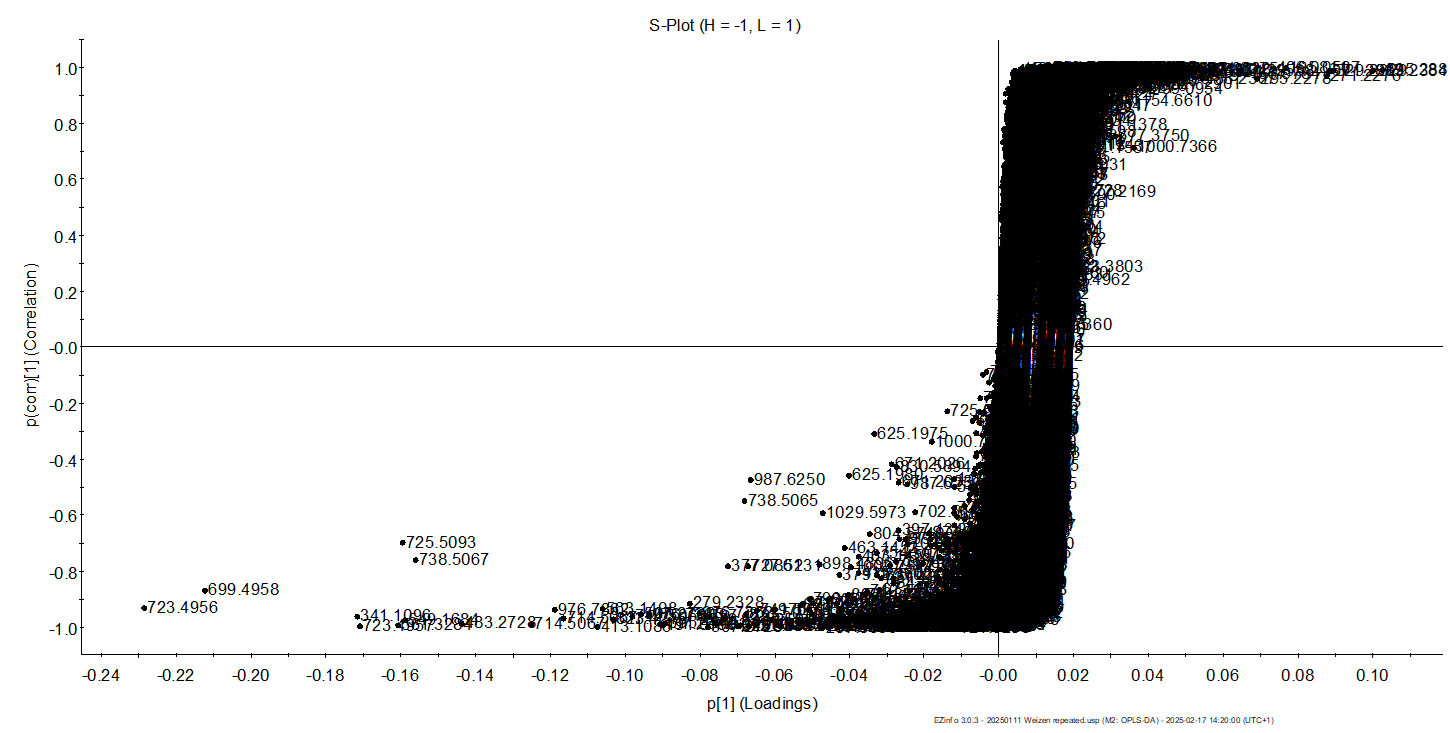
**

**Figure S27.** S-plot of wheat grain comparing metabolites from apogee H versus L grown indoors.

**References**

1 Ćurić, D., Karlović, D., Tušak, D., Petrović, B. & Đugum, J. Gluten as a standard of wheat flour quality. *Food Technology and Biotechnology* **4**, 353-361 (2001).

2 Freund, W. & Kim, M. 12 Determining the Baking Quality of Wheat and Rye Flour. (2006).

3 Laidig, F. *et al.* Breeding progress, environmental variation and correlation of winter wheat yield and quality traits in German official variety trials and on-farm during 1983–2014. *Theoretical and Applied Genetics* **130**, 223-245 (2017).

4 Mesdag, J. Variations in the protein content of wheat and its influence on the sedimentation value and the baking quality. *Euphytica* **13**, 250-261 (1964).

5 Olaerts, H., Vandekerckhove, L. & Courtin, C. M. A closer look at the bread making process and the quality of bread as a function of the degree of preharvest sprouting of wheat (Triticum aestivum). *Journal of Cereal Science* **80**, 188-197 (2018).

6 Bodor, K., Szilágyi, J., Salamon, B., Szakács, O. & Bodor, Z. Physical–chemical analysis of different types of flours available in the Romanian market. *Scientific Reports* **14**, 881 (2024).

7 Biel, W., Jaroszewska, A., Stankowski, S., Sobolewska, M. & Kępińska-Pacelik, J. Comparison of yield, chemical composition and farinograph properties of common and ancient wheat grains. *European Food Research and Technology* **247**, 1525-1538 (2021).

8 Högy, P. & Fangmeier, A. Effects of elevated atmospheric CO2 on grain quality of wheat. *Journal of Cereal Science* **48**, 580-591 (2008).

9 Maningat, C. C., Seib, P. A., Bassi, S. D., Woo, K. S. & Lasater, G. D. in *Starch* 441-510 (Elsevier, 2009).

10 Piironen, V., Lampi, A.-M., Ekholm, P., Salmenkallio-Marttila, M. & Liukkonen, K.-H. in *Wheat: chemistry and technology* 179-222 (AACC International, 2009).
